# Decoupling and Cloaking of Rectangular and Circular Patch Antennas and Interleaved Antenna Arrays with Planar Coated Metasurfaces at C-Band Frequencies—Design and Simulation Study

**DOI:** 10.3390/s24010291

**Published:** 2024-01-03

**Authors:** Shefali Pawar, Doojin Lee, Harry Skinner, Seong-Youp Suh, Alexander Yakovlev

**Affiliations:** 1 Department of Electrical and Computer Engineering, University of Mississippi, University, MS 38677-1848, USA; 2 Department of Electrical, Electronic, Control Engineering, Changwon National University, Changwon 51140, Republic of Korea; doojin.lee@changwon.ac.kr; 3 Intel Corporation, Hillsboro, OR 97124, USA; harry.g.skinner@intel.com (H.S.); seong-youp.suh@intel.com (S.-Y.S.)

**Keywords:** cloaking, coated metasurfaces, microstrip patch antennas, mutual interference, reduction of mutual coupling

## Abstract

An electromagnetic cloaking approach is employed with the intention to curb the destructive effects of mutual interference for rectangular and circularly shaped patch antennas situated in a tight spacing. Primarily, we show that by coating the top surface of each patch with an appropriately designed metasurface, the mutual coupling is considerably reduced between the antennas. Furthermore, the cloak construct is extended to a tightly spaced, interleaved linear patch antenna array configuration and it is shown that the coated metasurfaces successfully enhance the performance of each array in terms of their matching characteristics, total efficiencies and far-field realized gain patterns for a broad range of beam-scan angles. For rectangular patches, the cloaked Array I and II achieve corresponding peak total efficiencies of 93% and 90%, in contrast to the total efficiencies of 57% and 21% for uncloaked Array I and II, respectively, at their operating frequencies. Moreover, cloaked rectangular Array I and II exhibit main lobe gains of 13.2 dB and 13.8 dB, whereas uncloaked Array I and II only accomplish main lobe gains of 10 dB and 5.5 dB, respectively. Likewise, for the cloaked circular patches, corresponding total efficiencies of 91% and 89% are recorded for Array I and II, at their operating frequencies (uncloaked Array I and II show peak efficiencies of 71% and 55%, respectively). The main lobe gain for each cloaked circular patch array is approximately 14.2 dB, whereas the uncloaked Array I and II only achieve maximum gains of 10.5 dB and 7.5 dB, respectively.

## 1. Introduction

In view of the relentless explorations and investigations conducted by researchers and scientists over the span of the past few decades, the tantalizing aspect of invisibility is no longer an imaginary concept. Remarkably, the advent of metamaterials and metasurfaces has facilitated the development of electromagnetic invisibility, especially with regard to antennas as one of its most successful applications. We know that phased array antennas are growing in popularity due to numerous appealing aspects, such as high gain, high directivity, beam control, and ease of manufacturing, among others. Naturally, antenna arrays are expected to play a critical part in fulfilling the ever-increasing demand for channel requirements in radar and wireless communications services, which may require the deployment of crowded array systems in a very compact area. However, contrary to our expectation, such a dense system may exhibit performance degradation, owing to the cross-coupling between the antennas placed so close together. Consequently, several approaches have been developed in the last few decades to achieve electromagnetic cloaking, especially with respect to antennas, to make it undetectable to the surrounding sensors and thus immune to electromagnetic interference over a desired frequency range. The various techniques that have been reported include plasmonic cloaking [1,2,3,4,5,6], transformation-based cloaking [7,8,9,10,11], and transmission line networks [12,13,14], among others. Now, each of these prominent cloaking techniques has been shown to successfully induce electromagnetic invisibility; however, they have their own sets of advantages and disadvantages. An important limitation of the transformation-based and transmission line cloaking methods is that they are based on the electromagnetic isolation of the cloaked object (the concealed object is unable to transmit or receive electromagnetic energy), which means that they are impractical for sensing or antenna applications. In addition, these techniques are known to utilize bulk volumetric metamaterials and so might prove cumbersome for systems with minimal space capacity. As a possible solution to this problem, the mantle cloaking method was put forth at microwave frequencies [15,16,17,18] (simple, ultra-thin patterned metallic surfaces are employed to bring about cancellation of the dominant scattering mode). Moreover, it is important to note that mantle cloaks tend to induce electromagnetic invisibility by employing metasurfaces that cancel out the fields scattered by the intended object. This implies that the concealed object, in fact, is not electromagnetically isolated from the surrounding environment, which makes the mantle cloaking method a perfect candidate for sensing and/or antenna applications. A detailed and extensive review of the most renowned techniques used to obtain electromagnetic invisibility is presented in [19]. The implementation of the mantle cloaking approach has also been carried out at low-terahertz (THz) frequencies using graphene-based metasurfaces [20,21]. A vital application of the mantle cloaking method has been presented in [22,23], wherein the cloaks are demonstrated to overcome the mutual blockage between tightly placed antennas. The application of mantle cloaks to various cylindrical configurations has facilitated the cloaking of freestanding strip dipoles [24], planar microstrip monopole antennas [25], Yagi–Uda antennas [26], and, very recently, slot antennas [27]. These specially modeled mantle cloaks also bring about a cloaking effect amongst the neighboring antennas, such that they do not perceive each other [28,29,30]. In a similar fashion, at low-THz frequencies, graphene-based metasurface cloaks are used to reduce mutual interactions between planar antennas [31]. Additionally, in [32,33], wideband cloaking using mantle cloaks has been achieved for microstrip monopoles.

We should mention here that, besides cloaking techniques, several other mechanisms have been reported—for instance, electromagnetic band-gap structures (EBGs) [34,35] and parallel coupled line resonators [36]—to demonstrate the reduction of mutual coupling in patch antennas and arrays. The decoupling and suppression of cross-band scattering in antennas is also achieved through various devices and methods, as described in [37,38,39,40,41]. Besides this, in lieu of traditional metal antennas, efforts have been focused on dielectric resonator antennas (DRAs), especially for high micro-wave and millimeter-wave spectrum applications [42,43,44]. However, this is beyond the scope of our study, wherein we deal with the reduction of mutual coupling and scattering suppression in the case of microstrip patch antennas operating at two distinct frequencies.

To further emphasize the applicability of the mantle cloaking mechanism, studies [45,46,47] have been reported wherein circuit-loaded metasurfaces are shown to achieve waveform-selective invisibility. Here, the waveform-selective cloaking devices make an antenna either invisible or visible for either short pulses or continuous waves, thus leading to novel invisibility devices with advanced functionalities. The mantle cloaking approach has also been deployed for the cloaking of electrically large objects [48], and, recently, a novel cloaking technique was proposed in [49], wherein a planar metasurface for the cloaking of a free-standing bow-tie antenna and its various array configurations is presented. The relative simplicity of the metasurface design and the fact that it is capable of cloaking the entire surface of the bow-tie antenna (half-wavelength structure) motivated us to attempt to extend this cloaking principle to other half-wavelength antennas—for instance, simple patch antenna configurations. As a part of our recently published works, in [50], we have successfully demonstrated (through simulation results) the cloaking of equilateral triangle patch antennas and their array structures.

In this paper, we have targeted the very popular and widely used microstrip patch antenna systems—rectangular and circularly shaped patch antennas—along with their array configurations. Thus, taking inspiration from the design methodology in [49], we have devised a metasurface structure that facilitates the cloaking of these practical patch antennas. For each of the commonly shaped microstrip antennas, firstly, we establish the decoupling and cloaking effects for two patches placed in close proximity. We demonstrate that when the top surface of each patch antenna is coated by the specially tailored metasurface, they are decoupled (the effects of mutual coupling are nullified) from each other and the restoration of their radiation patterns is also observed, as if they were functioning in an isolated fashion (despite the two patches being confined close to each other). We then extend the cloak design to a one-dimensional linear interleaved array configuration of these patches and effectively demonstrate efficient array performance, which means that the dimensional area initially established for one array is now perfectly capable of accommodating two separate phased arrays, possibly leading to applications with compact space utilization. We would like to emphasize the novelty of our design, which sets it apart from other metasurface-based cloaking devices. To the best of our knowledge, in the reported literature [22,23,24,25,26,27], the cloaking structures employ dielectric substrates that are either circular or elliptical in shape, and the metallic elements are embedded periodically along the circumference of these substrates. It is an extremely arduous task to fabricate these metasurfaces, making the practical realization and the experimental verifications of such designs much more difficult. Moreover, these reported metasurfaces are required to maintain sub-wavelength cross-sectional dimensions as an important design constraint and cannot be used to cloak large antenna surfaces. Our proposed structure, on the other hand, is a simple planar design placed directly on top of the patch antenna, which makes it a more practical construct and hence more favorable from a fabrication point of view. Another important distinguishing characteristic of our design is that it covers (is coated on) the entire patch antenna’s surface area (whose resonant length is half the wavelength for a particular frequency in the dielectric medium), essentially achieving cloaking for an electrically large antenna surface. A very abridged version of our work related to the rectangular patch arrays is presented in our two-page conference paper [51], wherein the concept of coated metasurfaces for the cloaking of interleaved patch arrays is only briefly introduced. This article, however, presents a much more comprehensive analysis and details an exhaustive approach in order to justify the capabilities of the designed cloaks, not only for rectangular patches but also for circular patches. The design modeling, along with the numerical full-wave simulation results presented here, is obtained with the CST Microwave Studio [52].

The paper is sectioned as follows: Section 2 consists of the schematic figures and describes the design process for a module of two rectangular patch antennas designed to operate at different frequencies (uncloaked as well as cloaked structures); this is followed by Section 3, which details the simulation results, showcasing the decoupling and cloaking effects of the coated metasurface for the two rectangular microstrip antennas. Section 4 deals with the schematic configurations and simulation results for the interleaved rectangular patch arrays. In this section, we also highlight each array’s efficient beam scanning capabilities. In Section 5, we introduce circular patch antenna systems and demonstrate the decoupling and cloaking effects of the corresponding coated cloak structures for the two patches placed in close proximity. Furthermore, we exhibit the cloaking functionality of the metasurfaces on the one-dimensional interleaved array of the circular patches. Finally, we present the conclusions and the references for our work.

## 2. Design of Planar Coated Metasurfaces for Rectangular Patch Antennas

Our ultimate goal is to develop an interleaved system of two distinct phased arrays (wherein the elements of each array are placed in extremely close proximity) without compromising either array’s performance. To achieve this, we initiate our investigation by considering a single module of two simple, coaxially fed patch antennas, Patch I and II, operating at frequencies $f_{1}=4.9$ GHz and $f_{2}=5.2$ GHz, respectively. These patch antennas are embedded on a dielectric substrate (length $L_{g}=57.55$ mm and width $W_{g}=43$ mm) with thickness h=1.8 mm and permittivity $\varepsilon_{r}=2.2$ (Rogers RT5880 was employed), backed by a ground plane. We should emphasize the reason for choosing two different frequencies for the patches. It is a well-known fact that the detrimental mutual interference effects are predominantly observed between closely placed antenna elements. This destructive interference effect is even more pronounced when the designated operating frequencies of the antennas are also close (hence the reason for choosing operating frequencies of the patches merely 300 MHz apart). Moreover, C-band frequencies are targeted so that our designed antenna systems can be utilized for 5G applications.

**Figure 1:**
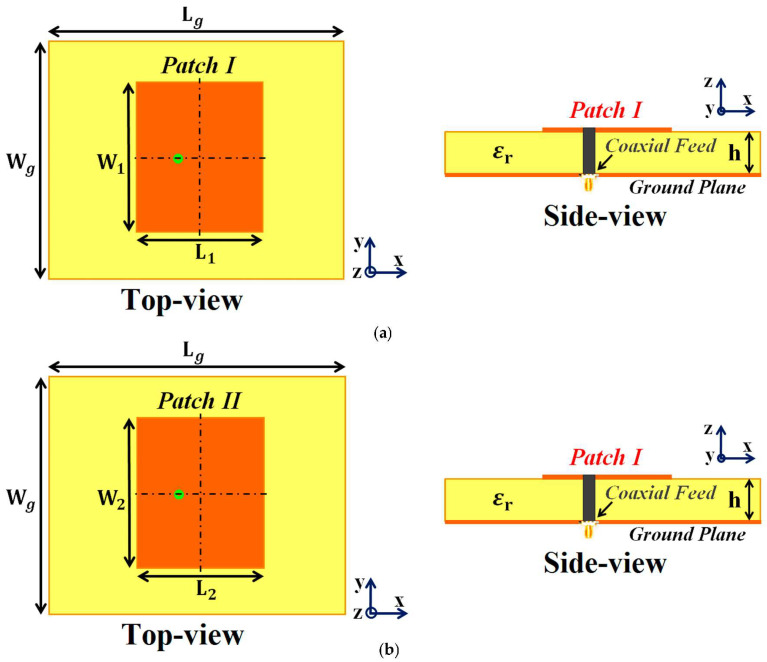
Schematic design configurations: (**a**) Isolated Patch I and (**b**) Isolated Patch II.

**Figure 2:**
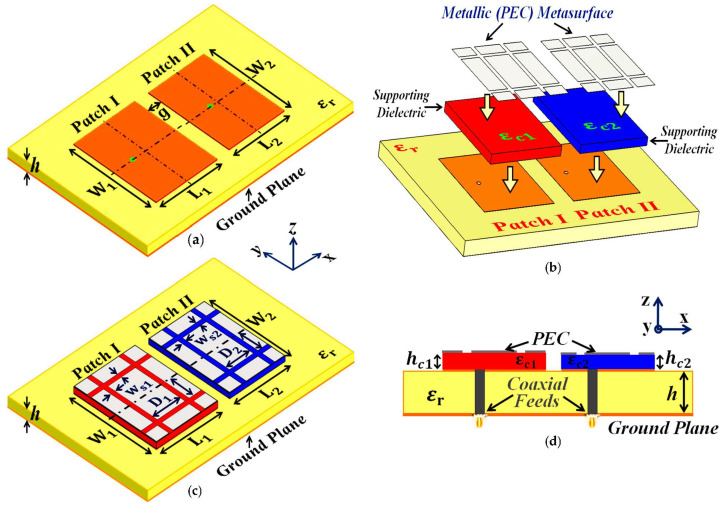
Schematics for (**a**) Uncloaked Patch I and II, (**b**) unfolded view of the cloak design for the patches, (**c**) Cloaked Patch I and II, and (**d**) side view of the cloaked rectangular patches, detailing the structural parameters of the coated metasurfaces.

Firstly, we design and examine each patch antenna separately and record their matching and radiation performance (see Figure 1a,b for the schematic figures). This is referred to as the *isolated* case, and we use the simulation results obtained from these scenarios as a template for comparison with the other cases. The parameters characterizing the patch antennas are $L_{1}=18$ mm, $W_{1}=23$ mm, $L_{2}=17.55$ mm, and $W_{2}=23$ mm. After cataloging the results obtained with the isolated patches, we proceed to situate the rectangular patches very close together on a single substrate (see Figure 2a; sub-wavelength separation is employed such that g=2 mm $\approx 0.037\;\lambda_{1}$, where $\lambda_{1}$ is the free space wavelength in relation to the frequency $f_{1}$). Since the separation g between them is extremely small, an undeniably strong mutual interference is caused in the near-field as well as the far-field of the structure, thereby resulting in the deterioration of the radiation properties of both the patches (simulation results are included in Section 3). Notice that in Figure 2a, the patch antennas are not covered with their corresponding cloaks yet and the mutual coupling effects are extensive in this particular scenario; thus, we term this the *coupled uncloaked* case. To address and curtail the detrimental impacts of mutual coupling, we devise a metasurface structure (also referred to as *cloaks*) for each patch antenna (refer to Figure 2b–d). It is our claim that these specifically engineered metasurface cloaks eradicate the adverse effects of mutual coupling (corroborated by the results shown in Section 3), essentially *decoupling* the rectangular patch antennas; therefore we aptly refer to this scenario as the *decoupled cloaked* case. The rationale behind using the term ‘metasurface’ for our proposed construct (despite being a finite and non-periodic structure) is that it is a 2D structure, wherein the dimensions of perfect electric conductor (PEC) patches as well as the gaps between these elements are sub-wavelength, which aligns with the description of a metasurface.

**Figure 3:**
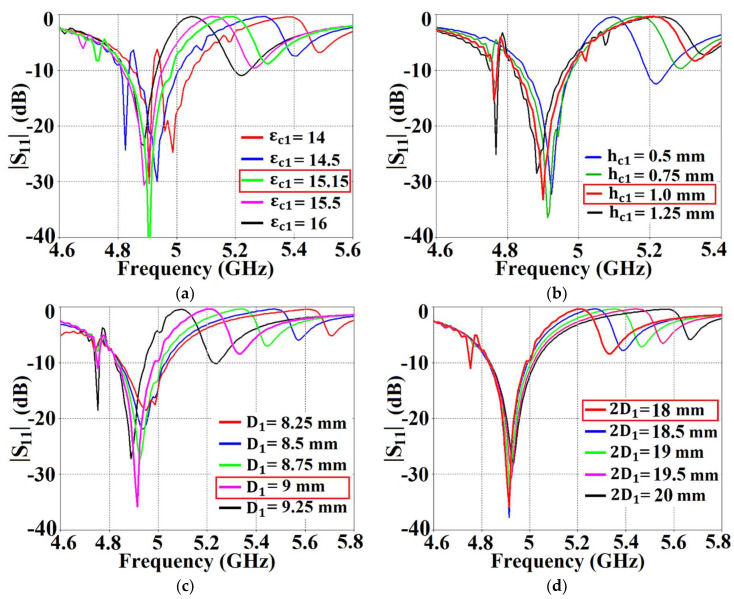
Parametric analysis using the reflection coefficients ($\left|S_{11}\right|$) for (**a**) relative permittivity $\varepsilon_{c1}$ of the supporting dielectric material, (**b**) thickness of the dielectric $h_{c1}$, (**c**) vertical slot placement $D_{1}$, and (**d**) horizontal slot placement ${2D}_{1}$ for the cloak design of Patch I.

In addition to this, our proposed structure, when integrated with the corresponding patch antenna, conveniently reduces its total radar cross-section (RCS) at the desired frequency (results are discussed in Section 3), thereby controlling and/or redirecting scattered electromagnetic fields around the antenna, thus demonstrating one of the many typical behaviors of a metasurface. We also refer to our structure as a ‘cloak’, owing to the fact that when the patch antenna is covered by it, the structure effectively makes the antenna ‘invisible’ to the electromagnetic fields at a particular frequency (an extremely narrowband frequency range, to be precise). We claim that the coated antenna becomes ‘invisible’ because, at the intended frequency, it does not reflect waves back to the source (as seen from the total radar cross-section plots in the following section) and also does not scatter waves in other directions. The deployment of our proposed metasurface cloaks entails the following steps. The top surface area of each patch antenna is first coated with a thin layer (sub-wavelength thickness) of high-dielectric-constant supporting material (thickness $h_{c1}=1$ mm, $h_{c2}=0.9$ mm, and relative permittivity $\varepsilon_{c1}=15.15$,$\;\varepsilon_{c2}=16.71$ for Patch I and Patch II, respectively), wherein the surface of each dielectric conforms thoroughly to the corresponding patch antenna. A PEC surface is then placed directly on top of each of these dielectrics. Note that thin slots are incorporated into this PEC surface, so that the resulting surface resembles an assembly of 2D sub-wavelength PEC patches, separated by extremely small gaps (slot widths are $w_{s1}=w_{s2}=0.75$ mm and spacing between the slots is $D_{1}=9$ mm and $D_{2}=9.3$ mm). The rationale behind choosing this particular design for our metasurface can be explained by the following argument. An important characteristic of the metasurface is that it does not perturb the radiation characteristic of its corresponding patch at the antenna’s operating frequency (resonance frequency). This indicates that the metasurface should be chosen such that it will mimic the behavior of the current on the patch antenna at the resonance frequency. Thus, metallic (PEC) patches are a natural choice. At the same time, we are concerned with making the metasurface ‘frequency-selective’, such that the scattering cancellation behavior is apparent only at a required specific frequency (termed the cloaking frequency, which is essentially the operating frequency of the neighboring patch antenna). For this purpose, small gaps are introduced between the PEC patches to create a situation similar to the periodic arrangement of metallic elements. Although the construct itself is rather straightforward, the design process is not analytically trivial. We endeavored to support our design with some form of analytical modeling in order to offer more rigorous theoretical explanations. In this regard, one of the sophisticated and popular means that we considered was the ‘generalized sheet transition conditions’ (GSTCs) for the modeling of metasurfaces in reference to cloaking. However, we quickly realized that there were certain restrictions in applying GSTCs to our design. First and foremost, this approach requires the metasurface to be electrically large (several wavelengths); however the metasurface in our design is restricted to the dimensions of its corresponding patch antenna (the resonant length of the patch antenna is half the operating wavelength in the dielectric medium). Additionally, in our designs, we observed that the patch antenna itself was strongly coupled to the metasurface, mainly because of the deeply sub-wavelength separation between the antenna and the metasurface. This involves higher-order mode coupling, which further complicates the matter as far as theoretical and analytical modeling is concerned. As such, although there are some highly sophisticated analyses available in the modeling of metasurfaces, we were not able to identify a satisfactory analytical model for our design. Nevertheless, we will continue this endeavor as part of our future work. At present, since we do not have a clear analytical model for our structure, parameterization is required in our design. Thus, rigorous parametric analysis was carried out to determine the optimum set of design parameters (i.e., varying one design parameter at a time within a certain range). To provide an insight into the steps undertaken to determine the cloak design constraints, several results are demonstrated through reflection coefficient plots ($\left|S_{11}\right|$) in Figure 3, showing the parametric study of the design specifications that are paramount to our metasurface configuration. Through the comprehensive analysis, we detected that among the many design parameters, the relative permittivity value of the supporting material (Figure 3a), the thickness of these dielectric materials (Figure 3b), and the placement of the vertical and horizontal slots (Figure 3c,d, respectively) on the PEC play a crucial role in bringing about the decoupling and cloaking effects at an intended frequency. For the sake of brevity, we have only included the analysis for the cloak design of Patch I. Here, we should remark that there are two frequencies of interest that are taken into consideration when designing a cloak for its corresponding patch antenna—namely, the *resonance frequency* and *cloaking frequency* (these frequency terms are used throughout the article). The resonance frequency is the frequency whereby an antenna is well matched with its input impedance (the reflection coefficient $\left|S_{11}\right|\leq 10$ dB) and is generally a good radiator at this frequency. On the other hand, the cloaking frequency indicates the frequency at which we are interested in observing the decoupling and cloaking effects. Typically, the reflection coefficient $\left|S_{11}\right|\approx 0$ dB at the cloaking frequency; consequently the antenna ceases to be an efficient radiator at this frequency. In our configurations, the cloaking frequency of one patch antenna is targeted at the resonance frequency of the other patch in its vicinity. For instance, let us consider Patch I. Patch I is designed to radiate at 4.9 GHz; thus, the resonance frequency of Patch I is 4.9 GHz. Subsequently, the cloaking frequency for Patch I should be targeted at 5.2 GHz (which is the resonance frequency of Patch II). Similarly, the resonance and cloaking frequencies for Patch II are 5.2 GHz and 4.9 GHz, respectively. In general, from Figure 3, we can see that although there are minimal deviations in the resonance frequency of Patch I (i.e., $f_{1}=4.9$ GHz), a more apparent tuning effect is observed for the frequencies where Patch I becomes unmatched (i.e., $\left|S_{11}\right|\approx 0$ dB).

**Figure 4:**
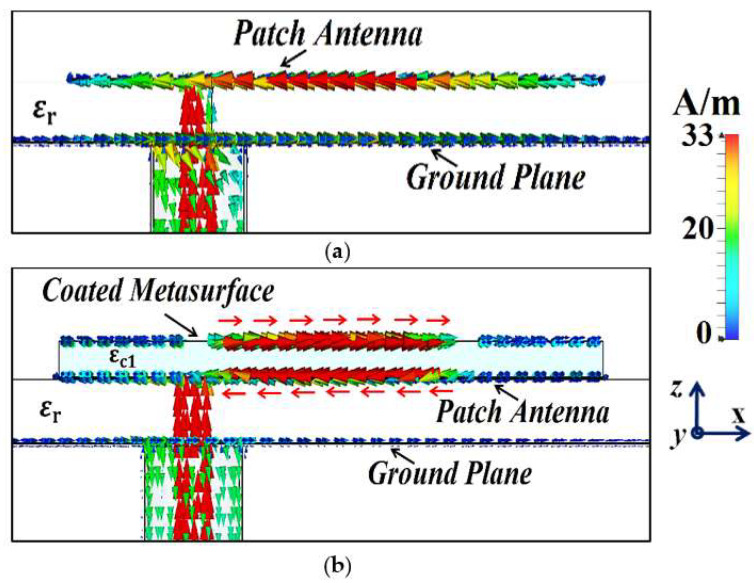
Cross-sectional view of the surface currents: (**a**) uncloaked and (**b**) cloaked Patch I at the cloaking frequency.

In Figure 3a, as the dielectric permittivity of the supporting material for the metasurface increases, the cloaking frequency steadily shifts to lower values. With the increase in the thickness of the supporting material, the cloaking frequency is seen to progress to higher values, apparent from Figure 3b. In regard to the placement of the slots on the PEC surface, the cloaking frequency decreases with the increase in vertical slot separation, whereas it is seen to increase with the increase in horizontal slot separation (see Figure 3c,d, respectively). As mentioned above, for Patch I, the frequency at which we wish to observe the decoupling and cloaking effects, i.e., the cloaking frequency, is set at $f_{2}=5.2$ GHz. Thus, as shown in the figures, the following values were chosen for the cloak design of Patch I: dielectric permittivity of the supporting material $\varepsilon_{c1}=15.15$, thickness $h_{c1}=1$ mm, vertical and horizontal slot separations $D_{1}=9$ mm and $2D_{1}=18$ mm. Through similar parametric investigations, appropriate values for the cloak design of Patch II were determined. Based on our deductions, the specific arrangement of our metasurface, employing the abovementioned parameters, caused the surface currents on the PEC to be routed in the direction opposite to that of the currents on the patch antenna surface, essentially giving rise to anti-phase surface currents. As an illustration, we present the cross-sectional view of the surface currents on an uncloaked and a cloaked antenna in Figure 4 (we consider the uncloaked and cloaked Patch I). It is obvious from Figure 4b that the direction of the currents on the planar coated metasurface is opposite to that of the surface currents on the patch antenna (to provide a clearer view, we highlight the routes of the currents on both the surfaces with red arrows). Analogous behavior of the surface currents is observed in the case of Patch II (results are not presented to avoid repetition). We believe that the fields induced by these anti-phase currents are ultimately responsible for the cancellation of the scattered fields by the targeted antenna at the intended cloaking frequency, in turn achieving the desired decoupling and cloaking effects.

A significant feature of our metasurface design is the use of high-dielectric-constant supporting materials ($\varepsilon_{c1}=15.15$ and $\varepsilon_{c2}=16.71$). The justification for the exploitation of such high dielectric permittivity values is given through the following observations and arguments. Firstly, in our proposed design, the PEC elements placed on the supporting dielectric materials act as a reactive load, effectively shifting the resonance frequency of the respective patch antenna to a slightly higher value. Now, the dielectric material for the metasurface acts as a superstrate to the patch antenna. The function of this superstrate is such that it marginally lowers the resonance frequency of the antenna. Thus, the high-permittivity supporting materials balance out the frequency tuning caused by the PEC elements of the metasurface, essentially ensuring that the resonance frequency of the respective patch antenna remains unaltered. Another interesting observation related to higher-permittivity materials is at the cloaking frequency of a patch (in terms of the reflection coefficient, $\left|S_{11}\right|\approx 0$ dB at this frequency). As seen from Figure 3a, the higher the relative permittivity, the lesser is the cloaking frequency. Here, the cloaking frequency for Patch I is set at 5.2 GHz, and, to achieve decoupling and cloaking effects at this frequency, a higher permittivity value is essential. If the cloaking effects were to be accomplished at some other higher frequency level, lower-dielectric-constant materials could have been employed. Regarding the role of dielectric permittivity in achieving the decoupling effects at a particular cloaking frequency, this can be understood as follows. As mentioned before, the proposed metasurface generates an anti-phase current on its surface. Now, the metallic (PEC) elements in our metasurface have a reactive surface impedance associated with it, and in order to produce the desired anti-phase surface currents, these metallic elements must acquire an appropriate surface impedance value at the intended frequency. We perceive that this is where the supporting dielectric material plays its role; a specific dielectric constant is required to achieve the necessary surface impedance of the metasurface so as to bring about the cloaking effects at a particular frequency.

We note that the investigations presented in this article are predominantly simulation-based. However, as a means of assuring the reliability of our results, we present the analysis of our cloak’s functionality from two fundamentally different perspectives—one as an ‘antenna problem’, wherein we scrutinize the performance of the uncloaked and cloaked antennas in terms of their radiation properties, matching characteristics, and total efficiencies (all of which are presented in Section 3), while the other we perceive as a ‘scattering problem’ in the presence of plane wave excitation. We investigate the behavior of the cloaked antennas through total radar cross-section (RCS) plots and electric field (E-field) contour plots at the respective cloaking frequencies of the rectangular patches (shown in Section 3). In the near future, to further support our claims, it is our goal to gather experimental verifications for our proposed design. As indicated above, the dielectrics used as supporting materials in the metasurface cloak design possess high permittivity values. At present, to be able to fabricate such substrates with low loss tangents, we are seeking reliable materials that can satisfy the high permittivity requirement and are considering the use of polymers infilled with ceramic materials so as to form a polymer composite. However, ceramic material compositions pose several difficulties, such as high costs for tools and challenging implementation, such as the requirement for high-temperature processing to achieve the desired structural integrity and also agreeable dielectric properties. As an alternative, we are also considering ‘additive manufacturing’, also known as 3D printing, and striving to gain access to a sophisticated industrial-grade 3D printing machine. A few options have been considered: the versatile fused filament fabrication (FFF) printer ‘Industry F421′ by 3DGENCE and the stereo lithography appearance (SLA) 3D printer ‘Lite600′ by UnionTech, among others. However, at present, we do not have access to such a sophisticated and expensive machine. We are working on securing funds for the same. We are also researching the optimum printing strategy with regard to a printer’s reliability in generating dielectric materials. The main process parameters, such as the printing speed, layer height, and material infill, are crucial in determining the impact on the relative permittivity values and loss tangents of the resulting printed samples [53], so as to avoid discrepancies in the measured results. Although, currently, this is proving to be an impediment from the fabrication point of view, we are confident that, in the near future, we will be able to manufacture reliable, high-dielectric-constant materials that are suitable for our designs, which in turn will facilitate experimental verifications for our configurations. Moreover, in our simulation models, we have employed supporting dielectric materials with a loss tangent (tan δ) of $9\times 10^{-4}$ and thermal conductivity as low as $0.2\;Wm^{-1}K^{-1}$. Through simulations, we have observed that materials with a tan δ as high as $5\times 10^{-3}$ are still tolerable but a further increase in the loss tangents destroys the decoupling and cloaking behavior of the metasurface. In addition, since we have considered the sub-6 GHz frequency range, the polarization effects of the dielectric materials are negligible and do not affect the permittivity values.

## 3. Simulation Results Showcasing Decoupling and Cloaking of Two Rectangular Patch Antennas

Some important settings that were established in the CST simulation 2019 software in order to simulate the results are as follows. We selected an ‘Open (add space)’ boundary type for all x, y, and z axes (to emulate the free space condition, which is recommended for antenna problems). Taking the required frequencies into consideration, we opted for the simulation frequency range of 2 to 7 GHz and set the minimum distance of the boundary box from the antenna structure at a one-fourth wavelength corresponding to the frequency 4.5 GHz. Next, the mesh properties were set as follows: the maximum cell was determined by setting the ‘cells per wavelength’ value as 39 and 21 for the cases near to the model and far from the model, respectively, whereas the minimum cell was generated by setting the value of the ‘fraction of maximum cell near to the model’ at 21. Utilizing these settings, the total number of cells created by CST was 2,750,940. These settings are undoubtedly paramount in determining the accuracy of the simulated plots; however, these settings are not at all rigid and should be customized as per individual requirements for respective simulation models.

**Figure 5:**
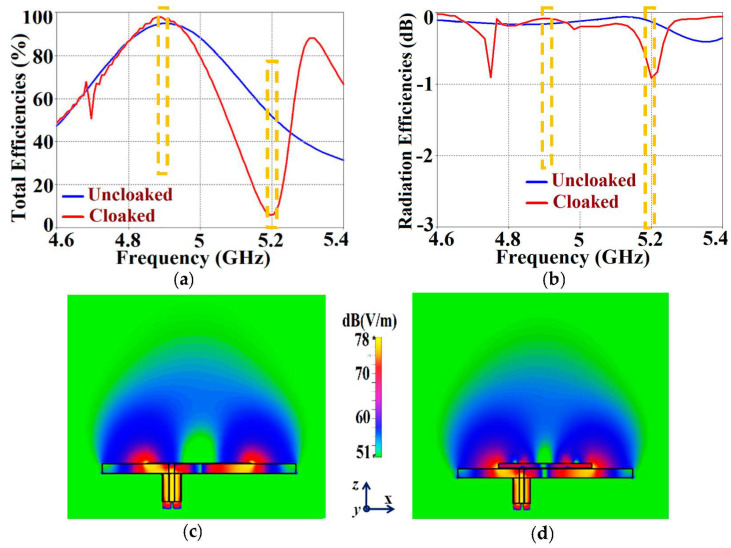
Plots for (**a**) total efficiencies, (**b**) radiation efficiencies, and electric field contours at $f_{1}=4.9$ GHz for (**c**) uncloaked and (**d**) cloaked Patch I.

First and foremost, we would like to highlight one of the most important traits of our proposed structures. It is remarkable that the metasurface coated on a patch does not affect any of the radiation characteristics at the resonance frequency of the antenna into which it is integrated; instead, its effects are observed at the frequency of the neighboring patch antenna. To better explain this phenomenon, we plot the total efficiencies along with the radiation efficiencies and E-field distributions for the uncloaked and cloaked Patch I in Figure 5. Let us observe the total efficiencies in Figure 5a: both the blue (for uncloaked Patch I) and red (for cloaked Patch I) curves show peak values of total efficiency at $f_{1}=4.9$ GHz (resonance frequency for Patch I). It is also seen from the red curve that the minimum value of approximately 6% occurs at $f_{2}=5.2$ GHz (cloaking frequency for Patch I). Looking at the radiation efficiencies in Figure 5b, it can be noted that the plots show analogous performance (to the total efficiencies). The blue and red curves show high radiation efficiency at $f_{1}=4.9$ GHz. In addition, the red curve attains a minimum value of the radiation efficiency (approximately −1 dB) at Patch I’s cloaking frequency ($f_{2}=5.2$ GHz). Thus, we effectively show that although Patch I is cloaked, it is still a very efficient radiator at its resonance frequency. However, the cloaked Patch I becomes an extremely poor radiator at its cloaking frequency. Moreover, comparing the E-field plots in Figure 5c,d, we can clearly see that the radiation behaviors of both the uncloaked and cloaked Patch I are almost identical. Consequently, we infer that the cloak construct coating Patch I allows the unaltered radiation of the antenna at 4.9 GHz but suppresses any scattering/radiation emanating at 5.2 GHz (in other words, Patch I is made electromagnetically invisible at its cloaking frequency, i.e., 5.2 GHz). Again, for the sake of brevity, we have not included the plots for Patch II, but very similar behavior is recorded for Patch II at its resonance and cloaking frequencies.

It should be noted that since the radiation efficiency is simply the ratio of the power radiated by an antenna to the power fed to the excitation port of the antenna, the power loss due to port impedance mismatch is not accounted for. On the other hand, the expression for total efficiency (as calculated by CST) takes into consideration the impedance matching characteristics (signified by the involvement of the reflection coefficient in the expression, i.e., either $S_{11}$ or $S_{22}$ from the S-parameter plots) along with the radiation properties (in terms of radiation efficiency) of the antennas. The total efficiency is computed as $\eta_{total}=\left[(1-{\left|\Gamma \;\right|}^{2})\eta \right]$, where $\eta_{total}$ signifies the total efficiency, Γ denotes the reflection coefficient ($S_{11}$ or $S_{22}$), and η denotes the radiation efficiency. Since the computational expression for the total efficiency encompasses both the matching characteristics and the radiation efficiencies of the corresponding antennas, henceforth, we display only the total efficiency plots.

**Figure 6:**
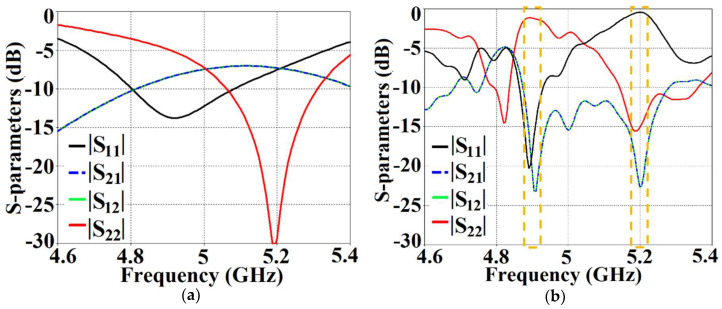
S-parameter plots for (**a**) coupled uncloaked and (**b**) decoupled cloaked rectangular patch antennas.

**Figure 7:**
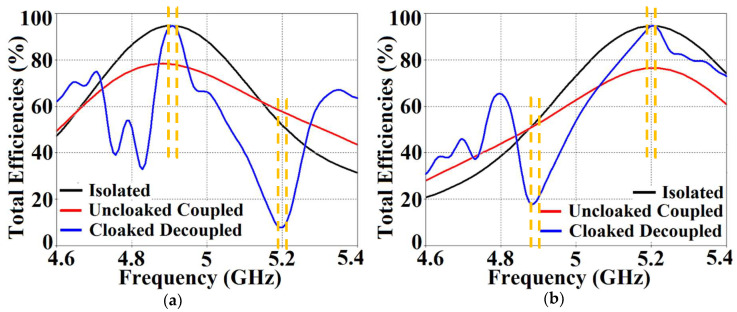
Total efficiencies when (**a**) Patch I is active and (**b**) Patch II is active.

**Figure 8:**
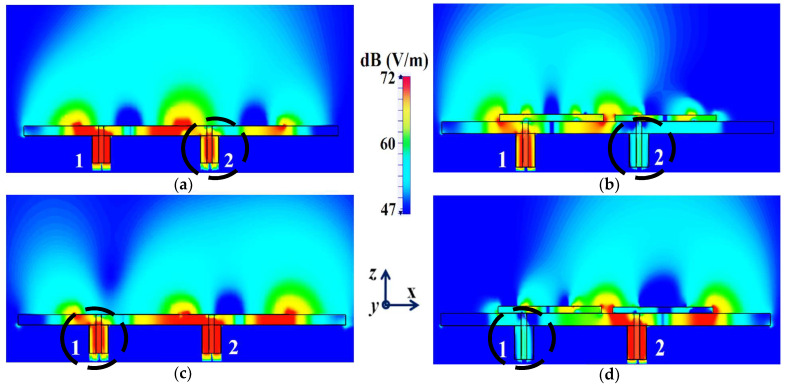
Electric field contours for the two rectangular patches placed close together: (**a**) coupled uncloaked (without cloaks) and (**b**) decoupled cloaked (with cloaks) cases, when Patch I ($f_{1}=4.9$ GHz) is active, and similarly for (**c**) uncloaked and (**d**) cloaked cases, when Patch II ($f_{2}=5.2$ GHz) is active.

As mentioned in the previous section, we have analyzed our design configurations from two different perspectives. Initially, we focused our investigations on the ‘antenna problem’ perspective (essentially indicating that we regard our structures as active devices) and we remark on the matching and far-field radiation aspects of the proposed system in the following analysis. As a part of this analysis, we present the S-parameter plots in Figure 6, along with the plots for the total efficiencies in Figure 7, followed by the E-field distribution contours in Figure 8 for tightly arranged rectangular patches (both uncloaked and cloaked configurations) to demonstrate the decoupling and cloaking effects of the coated metasurface. Recall that the coupled uncloaked case refers to the scenario in which the two patches are situated in a tight spatial arrangement (g=2 mm) and neither of the patches is coated with the metasurface cloaks. Naturally, the close proximity of the patch antennas introduces a considerable amount of mutual coupling between Patch I and II. This is evident from Figure 6a, where the coupling coefficients $\left|S_{12}\right|=\left|S_{21}\right|>-10$ dB at both $f_{1}$ and $f_{2}$. Please note that the green and dotted blue curves, indicating the mutual coupling levels in Figure 6, are overlapped completely and so they tend to appear as a single plot instead of the two curves that have been plotted. Accordingly, from Figure 6b, we can see that when the individual patches are cloaked by their respective metasurfaces, the magnitudes of the coupling coefficients ($\left|S_{12}\right|$ and $\left|S_{21}\right|$) clearly decrease; a reduction of almost 15 dB in $\left|S_{12}\right|$ and $\left|S_{21}\right|$ is recorded at $f_{1}$ as well as $f_{2}$. Additionally, from Figure 6b, notice that $\left|S_{11}\right|\approx -20$ dB at frequency $f_{1}$ and it increases to $|S_{11}|\approx 0$ dB at frequency $f_{2}$, indicating that Patch I is perfectly matched and, therefore, a good radiator at $f_{1}$, but it is completely unmatched and, hence, a poor radiating device at $f_{2}$.

In a similar fashion, $\left|S_{22}\right|\approx -15$ dB at frequency $f_{2}$ and then reaches $\left|S_{22}\right|\approx -1$ dB at frequency $f_{1}$, indicating that Patch II radiates excellently at $f_{2}\;$; however, it becomes a poor radiator at $f_{1}$. Here, we should comment on the narrowband response of our proposed structure. From the plots in Figure 6b, the impedance bandwidths (corresponding to $\left|S_{11}\right|=\left|S_{22}\right|<-10$ dB) at $f_{1}$ ≈1.24% and at $f_{2}$ ≈2.9%, making our designs very much frequency-specific (this narrowband behavior is observed for the cloaked configurations of both rectangular as well circular patches). This is mainly attributed to the fact that the cloaking structure utilizes a single metasurface element, and it is the nature of such resonant single metasurface cloaks to culminate in narrowband cloaking. Following the wideband cloaking method described in [32], we speculate that a stacked multi-layered planar metasurface may assist in improving the impedance bandwidth performance of our proposed cloaking process for the microstrip patch antennas. As such, we are actively involved in exploring such structures for wideband cloaking as a part of our future work. We also display the plots for the total efficiencies of the rectangular patch antennas in the isolated, coupled uncloaked and decoupled cloaked cases in Figure 7. Due to the presence of mutual coupling between the uncloaked patches, the total efficiency drops by approximately 18% and 20% for Patch I and II, respectively, at their corresponding resonance frequencies (compare the red curves in Figure 7a,b with the black curves to identify the decrease in the efficiency levels). On the other hand, for the cloaked scenario (illustrated by the blue curves in Figure 7a,b), the total efficiencies of each of the patches are seen to have recovered. It is worth noting that the restored efficiency levels are equal to the efficiencies recorded for the isolated cases, denoted by black curves in Figure 7, and although the total efficiency of a cloaked patch antenna remains unchanged at its own resonance frequency, it reduces significantly at the resonance frequency of the neighboring patch antenna. This supports our previous assertion that the patterned metasurface does not change the radiation aspects of the patch antenna on which it is coated; instead, its effect is evident at the cloaking frequency (which is the operating frequency of the other patch in its vicinity). Moreover, in Figure 8, we present an expanded view of the E-field plots for the closely placed rectangular patches (with and without cloaks). Through the E-field plots, we endeavor to visually represent the effects of mutual coupling on the corresponding ports and also on each antenna’s radiation behavior, in their uncloaked form. Subsequently, we show that when the patch antennas are coated by the planar metasurfaces (cloaked cases), the coupling effects are considerably minimized. In Figure 8a,b, Patch I (resonance frequency, $f_{1}=4.9$ GHz) is excited, keeping Patch II (resonance frequency, $f_{2}=5.2$ GHz) inactive.

**Figure 9:**
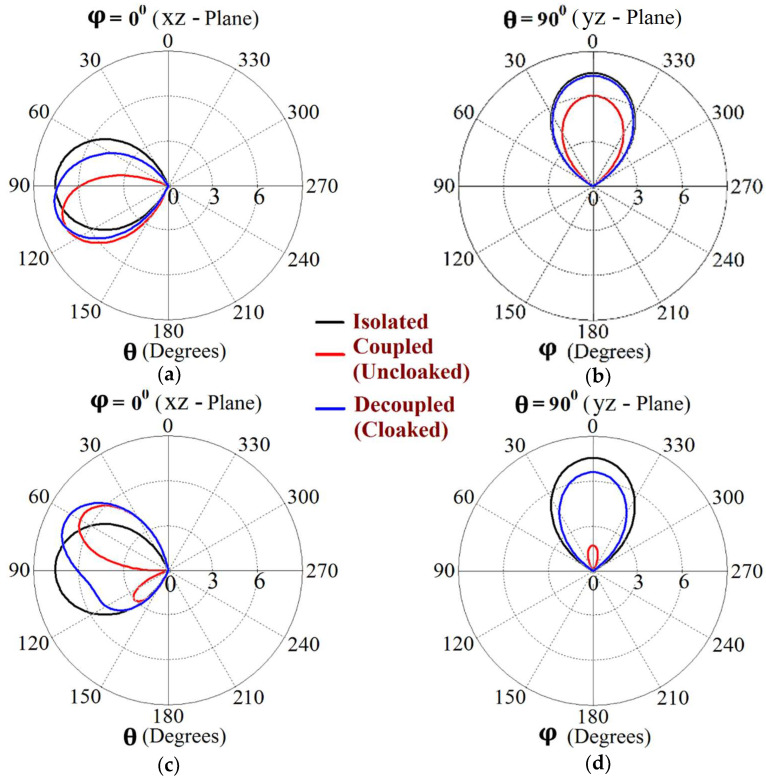
Realized gain patterns at (**a**)$\;\varphi =0^{{}^{\circ}}$, (**b**) $\theta =90^{{}^{\circ}}$ for Patch I (at frequency $f_{1}=4.9$ GHz), and at (**c**)$\;\varphi =0^{{}^{\circ}}$, (**d**) $\theta =90^{{}^{\circ}}$ for Patch II (at frequency $f_{2}=5.2$ GHz).

Now, we observe Figure 8a; for the uncloaked antenna models, mutual interference is obvious in the sense that power is seen to be coupled from the input port of Patch I (denoted as 1) to the neighboring patch antenna port (denoted as 2). This is deduced based on the high concentration of fields seen at port 2 (evident by the red colored region), despite the fact that Patch II is not active. On the other hand, when the metasurfaces are employed (cloaked case, see Figure 8b), power coupling from port 1 to 2 is greatly minimized, thus highlighting the decoupling behavior of the cloak structure. Similarly, Figure 8c,d correspond to the uncloaked and cloaked cases, respectively, when Patch II is excited and Patch I is passive. Following the above argument, analogous deductions can be made for Patch II. To further illustrate that the coated metasurfaces not only decouple the two antennas, but are also capable of restoring the far-field radiation behavior of individual patches, we present the polar plots for the realized gain patterns in Figure 9. The simulated main lobe gain of both the patches in the isolated scenario is around 7.5 dBi (recall that the isolated scenario represents the condition where measurement for each patch is carried out in the absence of the other and the radiation patterns for the same can be regarded as the ideal cases). We then compare the polar plots for the realized gain of the coupled (uncloaked) and decoupled (cloaked) patches. In Figure 9, the radiation patterns are plotted in the xz (also denoted as $\varphi =0^{{}^{\circ}}$) and yz (also denoted as $\theta =90^{{}^{\circ}}$) planes of reference for the patch antennas I and II at their corresponding resonance frequencies, i.e., $f_{1}=4.9$ GHz and $f_{2}=5.2$ GHz, respectively. For the coupled (uncloaked) case, a considerable distortion in the gain patterns for both Patch I and Patch II is apparent (observe the solid red curves in Figure 9). Nevertheless, it is obvious that the specifically tailored metasurfaces faithfully reinstate the gain patterns for both the patches (refer to the decoupled cloaked case shown by the solid blue curves in Figure 9), at both the planes of reference.

**Figure 10:**
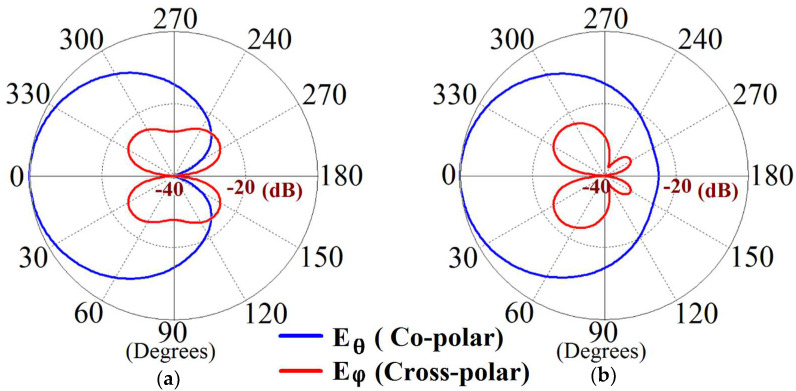
E-field plots showing co-polar and cross-polar radiations for the cloaked configurations of (**a**) Patch I at $f_{1}=4.9$ GHz and (**b**) Patch II at $f_{2}=5.2$ GHz.

Furthermore, we note the cross-polarization levels of our rectangular patch antennas. On the basis of our design configurations, the rectangular antennas are horizontally polarized, and our observations indicate that patch antennas I and II show cross-polarizations of approximately −24.2 dB and −22.7 dB at their respective resonance frequencies. Even when they are cloaked, the cross-polarization levels are maintained at −24.2 dB and −22.7 dB, for Patch I and II, respectively. This highlights the fact that the metasurface cloak does not interfere with the polarization levels of the antenna on which it is coated. We present the co-polar and cross-polar E-field radiation in the following Figure 10 for the cloaked Patch I and II at their respective resonance frequencies. Through the numerous simulation results demonstrated above, we emphasize the decoupling effect of our metasurface in the near-field and also highlight that the metasurface cloaks restore the far-field radiation patterns of each of the antennas, thereby emphasizing the cloaking behavior of the proposed structures.

**Figure 11:**
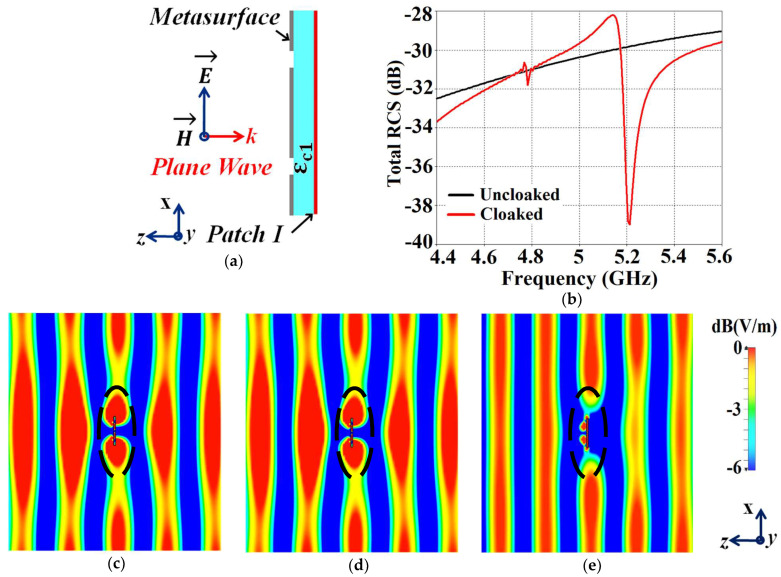
(**a**) Cross-sectional view of Patch I coated with the metasurface cloak, (**b**) total RCS plot for Patch I and E-field plots for (**c**) uncloaked Patch I at $f_{1}=4.9$ GHz, (**d**) cloaked Patch I at $f_{1}=4.9$ GHz, and (**e**) cloaked Patch I at $f_{2}=5.2$ GHz in presence of a normally incident TM polarized plane wave.

We now proceed to investigate the system as a ‘scattering problem’. Here, the patches coated with the cloak structures are essentially treated as passive devices and are bombarded by plane waves to study the scattering behavior of a patch antenna, with and without the cloak. For this purpose, we consider a transverse magnetic (TM) polarized plane wave and apply it to our design configurations to analyze their scattering performance. The detailed scattering aspects of Patch I are exhibited in Figure 11 (the design setup for the integrated system of the patch antenna coated with its corresponding metasurface in the presence of plane wave excitation is depicted in Figure 11a). Through the total RCS plot in Figure 11b, we demonstrate the scattering cancellation action of our proposed metasurface. Comparing the black and red curves for the uncloaked and cloaked Patch I, respectively, we see a significant decrease in the scattering width, which is evident from the 9 dB drop (approximately) recorded at Patch I’s cloaking frequency (i.e., $f_{2}=5.2$ GHz). Next, let us observe the behavior of the E-field around the uncloaked Patch I; considerable scattering is seen around the edges, shown by the red-colored region in Figure 11c. Let us compare this to the E-field plots for the cloaked Patch I. Figure 11d depicts the E-field contour plots for the cloaked Patch I at its resonance frequency (i.e., $f_{1}=4.9$ GHz). We can clearly see the scattering of the E-field around the cloaked structure (evident by the concentration of the red-colored region around the patch edges) and notice that this scattering behavior is almost identical to that illustrated in Figure 11c, for the uncloaked patch. This is added confirmation that the designed metasurface does not harm or alter the radiation/scattering behavior of the patch antenna at its resonating frequency. However, let us consider the cloaking frequency of Patch I ($f_{2}=5.2$ GHz), as illustrated in Figure 11e. At this frequency, the metasurface completely eliminates the scattering around Patch I (evident by the absence of red-colored regions around the patch edges) and the almost smooth passage of the E-fields through the patch is observed, suggesting that the electromagnetic fields cannot ‘see’ it. Thus, we deduce that Patch I is compelled to become electromagnetically invisible at 5.2 GHz. Similar arguments can be made for the case of cloaked Patch II; however, the corresponding results are not included here for brevity and to avoid repetition.

We summarize that the specifically designed cloaks not only improve the near-field characteristics (which essentially entails the decoupling of the antennas; in this section, the decoupling behavior is depicted through S-parameter plots, total efficiencies, and near-field E-field distribution plots), but also reinstate and improve the radiation properties of the antenna in the far-field (which we have demonstrated through the various polar plots). In addition, we have also showcased the total RCS plots and E-field plots, in the presence of a TM polarized plane wave excitation source, to highlight the scattering cancellation capability of the cloaks, which essentially accentuates the far-field cloaking functionality of the proposed metasurface. In the following section, we extend our metasurface design configuration to a linearly arranged, interleaved array of two rectangular patches.

## 4. Cloaking of the Interleaved Rectangular Patch Arrays

**Figure 12:**
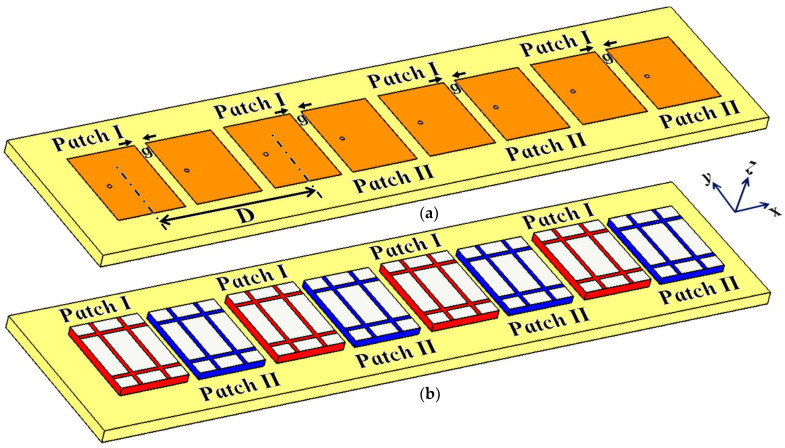
Schematic configurations of (**a**) uncloaked and (**b**) cloaked rectangular patch antenna arrays.

The cloak design specified in the aforementioned section is further extended to an interleaved array of patch antennas, wherein the module of the two patches (as described in Section 2) is linearly repeated in the direction of the x-axis and mounted on a single substrate with thickness h=1.8 mm and permittivity $\varepsilon_{r}=2.2$ (i.e., the Rogers RT5880 substrate is utilized). Refer to the design configuration shown in Figure 12. Accordingly, there are four elements each for Patch I and Patch II in the array system. All the Patch I elements form one array, which is why they are excited simultaneously, and they will be referred to as Array I henceforth. In a similar fashion, all the Patch II elements form the second array, which we term Array II. Each element of Array I is spatially separated by a distance of D=40 mm, whereas the elements of Array II are positioned alongside the elements of Array I at a distance of g=2 mm. Consequently, we have designed an interleaved system of two distinct arrays, essentially utilizing the same dimensional area that would have been employed for a single array. In a typical fashion, when the antennas are without their cloaks, the close proximity of these patch elements causes strong interference, which destroys the matching as well as radiation aspects of both the arrays. As such, as these two arrays are so closely packed, the neighboring antenna elements are strongly coupled, deteriorating the total efficiency as well as the gain of each participating array. Thus, contrary to the expected behavior of an array system, the efficacy of each array in this case is actually degraded. To improve the productivity of these arrays, we deploy the planar coated cloaks, as discussed in Section 2, to the respective rectangular patch antenna elements in our array configuration (illustrated in Figure 12b).

Following the CST simulation settings described in the first paragraph of Section 3, to obtain the following demonstrated results in the case of the interleaved rectangular arrays, we set the mesh cell properties as follows: the maximum cell is determined by setting the ‘cells per wavelength’ value at 27 and 21 for the cases near to the model and far from the model, respectively, whereas the minimum cell is generated by setting the value of the ‘fraction of maximum cell near to the model’ as 21. Thereby, the total number of cells created by the CST software using these settings is 2,863,714. Again, these settings are flexible and can be customized depending on the version of CST being used or as per the design requirements.

**Figure 13:**
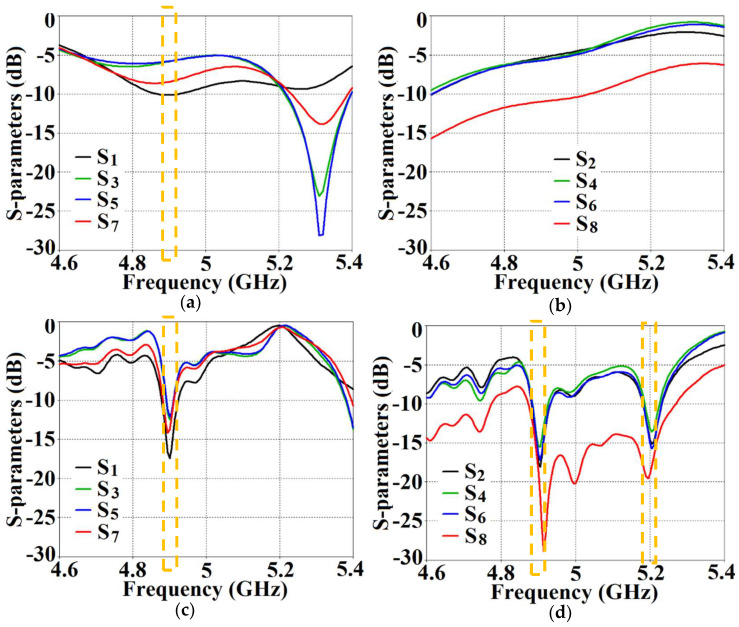
(**a**) Active reflection coefficients, (**b**) active coupling coefficients for uncloaked (coupled) Array I, (**c**) active reflection coefficients, and (**d**) active coupling coefficients for cloaked (decoupled) Array I (resonance frequency $f_{1}=4.9$ GHz).

**Figure 14:**
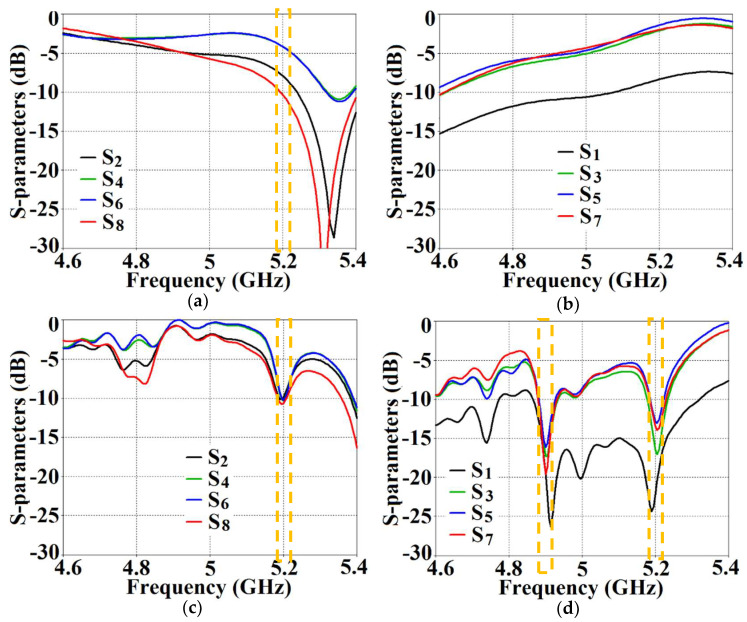
(**a**) Active reflection coefficients, (**b**) active coupling coefficients for uncloaked (coupled) Array II, (**c**) active reflection coefficients, and (**d**) active coupling coefficients for cloaked (decoupled) Array II (resonance frequency $f_{2}=5.2$ GHz).

With the intention to effectively demonstrate that the arrays are decoupled from each other, we present the isolation parameters (coupling coefficients) for the cloaked arrays and compare them with their uncloaked counterparts (Figure 13 and Figure 14). Additionally, we also show the reflection coefficients at the respective ports. Consider Figure 13, which depicts the isolation and reflection coefficients for the uncloaked and cloaked versions of Array I (the resonance frequency of this array is $f_{1}=$ 4.9 GHz). When Array I is made active, this means that ports 1, 3, 5, and 7 are excited. Therefore, when Array I is activated, the active reflection coefficients are observed at ports 1, 3, 5, and 7 (denoted as $S_{1}$, $S_{3}$, $S_{5}$, and $S_{7}$), whereas the active coupling coefficients are studied at ports 2, 4, 6, and 8 (denoted as $S_{2}$, $S_{4}$, $S_{6}$, and $S_{8}$). Evidently, from Figure 13a, the matching characteristics for uncloaked Array I are completely destroyed at the resonance frequency $f_{1}=$ 4.9 GHz. This is predominantly attributed to the high levels of mutual coupling arising due to the neighboring antenna elements; the isolation parameters shown in Figure 13b make this abundantly clear. It is fascinating to see that Array I (in the uncloaked form) not only becomes a non-radiator at its own resonance frequency but a tremendous amount of power coupling is evident at the neighboring array ports.

**Figure 15:**
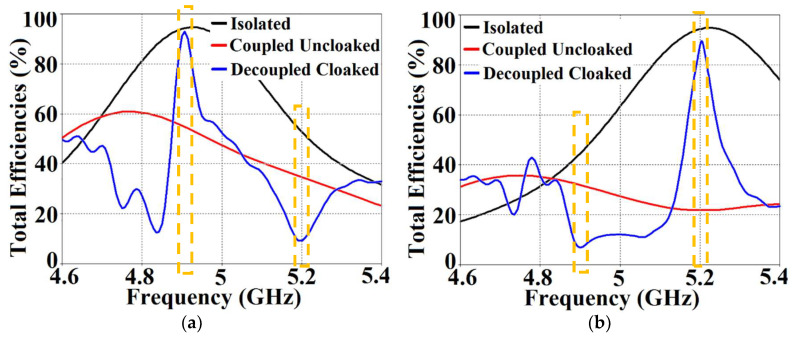
Plots for total efficiencies: (**a**) Array I (resonance frequency $f_{1}=4.9$ GHz) active and (**b**) Array II (resonance frequency $f_{2}=5.2$ GHz) active.

**Figure 16:**
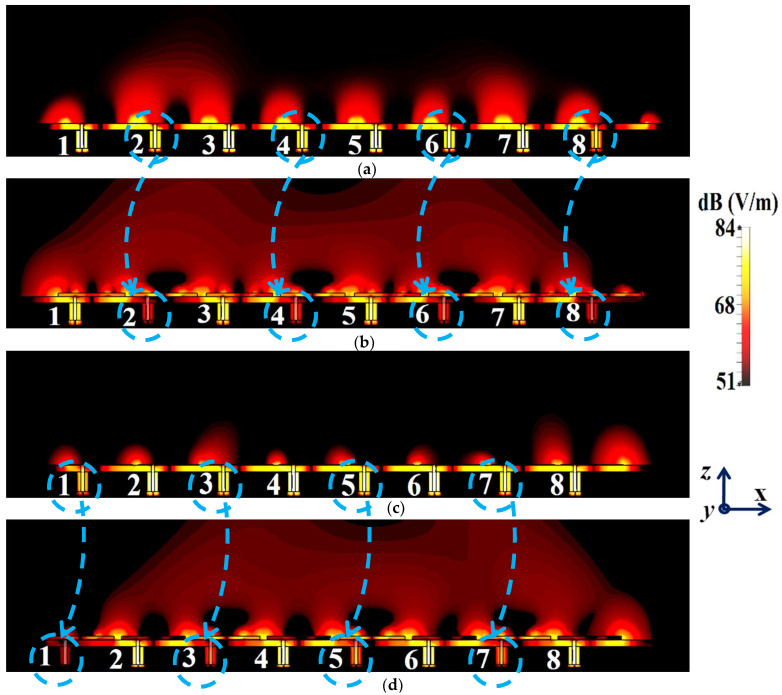
Electric field contours for (**a**) uncloaked and (**b**) cloaked patch antenna arrays when Array I (operating frequency, $f_{1}=4.9$ GHz) is active; (**c**) uncloaked and (**d**) cloaked patch antenna arrays when Array II (operating frequency, $f_{2}=5.2$ GHz) is active.

On the other hand, when the arrays are coated with our proposed metasurface design, the Array I elements are seen to be perfectly matched at 4.9 GHz in Figure 13c. Along with this, Array I is effectively unmatched at the neighboring array’s frequency, i.e., at $f_{2}=$ 5.2 GHz. This is credited to the fact that the coated metasurfaces greatly reduce the mutual coupling magnitudes (see Figure 13d) at each resonance frequency, essentially decoupling the neighboring array elements. Similar arguments and observations can be made when Array II is active. In this case, ports 2, 4, 6, and 8 are excited, which means that the active reflection coefficients (denoted as $S_{2}$, $S_{4}$, $S_{6}$, and $S_{8}$) are plotted at these ports and the isolation coefficients (denoted as $S_{1}$, $S_{3}$, $S_{5}$, and $S_{7}$) are plotted at ports 1, 3, 5, and 7 (refer to Figure 14). The decoupling and cloaking effects of our metasurface structures are also apparent in the plots for the total efficiencies and the E-field distributions, provided in Figure 15 and Figure 16, respectively. The total efficiencies are plotted for each array in the isolated scenario and coupled uncloaked and decoupled cloaked conditions. Referring to Figure 15a,b, it is clear that the total efficiency decreases remarkably for the uncloaked arrays, especially in the case of Array II (depicted by the solid red curves). A drop of approximately 30% is recorded for Array I and a significant 75% reduction is seen for Array II, which means that the matching and radiation aspects of Array II are completely destroyed.

For the cloaked arrays, however, the total efficiencies are largely recovered at their respective resonance frequencies (illustrated by the solid blue curves), almost emulating the efficiency values of the corresponding array in the isolated scenario (indicated by black curves). Once again, along with improving the total efficiency of each array at their respective resonance frequencies, the efficiency values attain negligible levels at the respective cloaking frequencies of the arrays (nearly reaching 10% at 5.2 GHz for Array I and 7% at 4.9 GHz for Array II). The E-field contour plots for our array arrangement are presented in Figure 16; this serves to provide additional validation of the decoupling functionality of the metasurface constructs.

In Figure 16a,b, a comparison of the field plots is shown for the uncloaked and cloaked scenarios, respectively, when Array I is active (ports 1, 3, 5, and 7 are excited simultaneously) and Array II is kept inactive. Similarly, field plots when Array II is active (ports 2, 4, 6, and 8 are excited) and Array I is passive are shown in Figure 16c,d. For the uncloaked coupled scenario, unmistakable coupling is apparent between the neighboring elements of the interleaved arrays, ultimately inhibiting the far-field radiation efficacies of each array. For instance, consider Figure 16a,b; here, Array I is active. Despite this, for the uncloaked case (Figure 16a), power coupling is noticeable at the input ports of Patch II, i.e., at ports 2, 4, 6, and 8. On the other hand, in Figure 16b, the coupling becomes almost negligible when the array elements are cloaked by the planar metasurfaces. Ports 2, 4, 6, and 8 are marked in Figure 16a,b to highlight the decrease in power coupling at these input ports, in turn ensuring a reduction in the unwanted mutual interference between the neighboring elements of the two arrays. A very similar behavioral pattern can be deduced from Figure 16c,d, where Array II is active. Therefore, we reiterate that when employing the specialized planar cloaks on the respective patch element, the coupling effects are significantly diminished in the near-field. Along with this, the restoration of the radiation patterns is noticed in the far-field, leading to a vast improvement in the overall radiation properties of each array.

### Beam Scanning

The coated metasurfaces are engineered with the sole purpose of enhancing the properties of each array such that they are comparable with the isolated array performance, which significantly increases the productivity of the array system as a whole, in spite of the crowded arrangement of the antenna elements.

**Figure 17:**
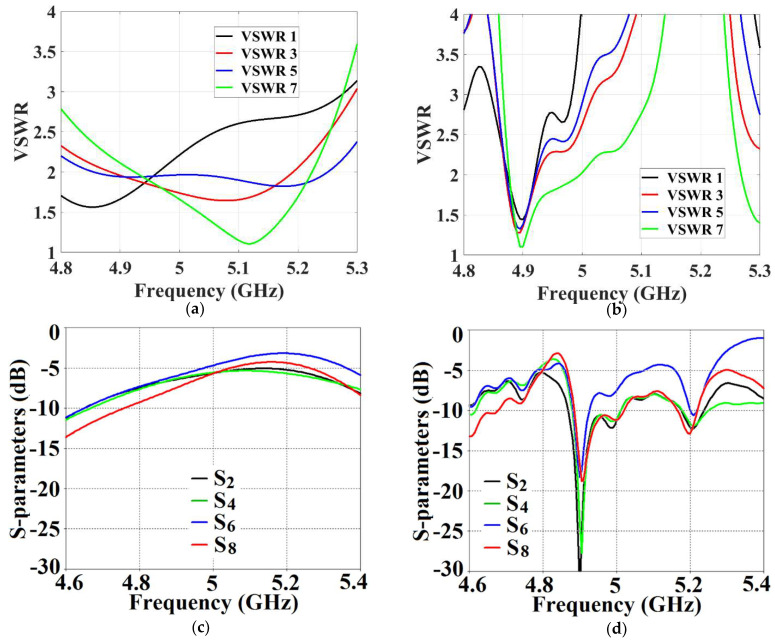
Active VSWR plots for (**a**) uncloaked coupled and (**b**) cloaked decoupled Array I ($f_{1}=4.9$ GHz), and isolation parameter plots for (**c**) uncloaked coupled and (**d**) cloaked decoupled Array I, at scan angle = $20^{{}^{\circ}}$.

**Figure 18:**
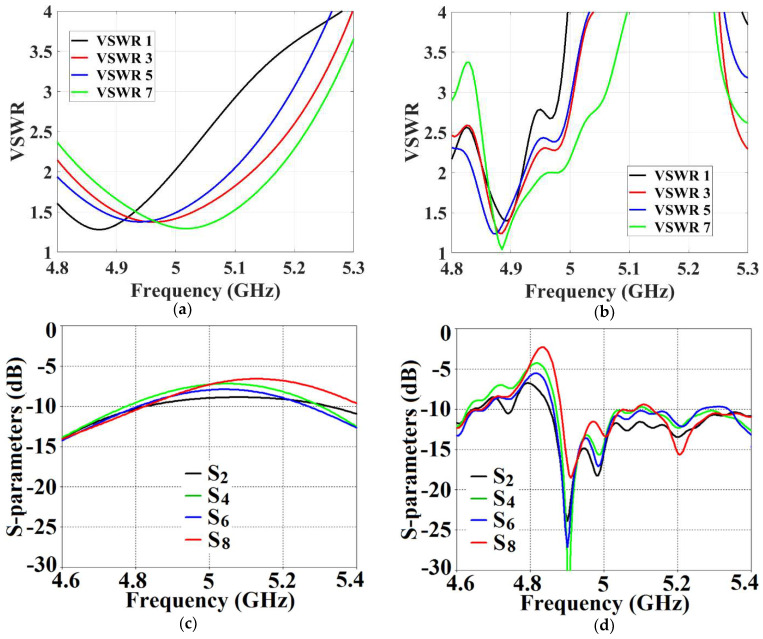
Active VSWR plots for (**a**) uncloaked coupled and (**b**) cloaked decoupled Array I ($f_{1}=4.9$ GHz), and isolation parameter plots for (**c**) uncloaked coupled and (**d**) cloaked decoupled Array I, at scan angle = $30^{{}^{\circ}}$.

This is achieved by ensuring that all the elements of one array are made invisible to (decoupled from) all the elements of the neighboring array; in turn, this means that the two arrays can operate as if they were isolated (operating independently) from each other. This forms the basis for enabling efficient beam scanning at various scan angles. We know that by exciting the corresponding port of a targeted array with proper phase shifts, a desired beam angle can be achieved (we used the well-known formula to calculate the required phase shift for the excitation signal that serves as an input for particular antenna elements of an array). Furthermore, we also determined the estimated range of beam scanning angles for our array configurations. It follows that both the designed arrays (Array I and II) have the ability to scan the beam from ${-45}^{{}^{\circ}}$ to $45^{{}^{\circ}}$ (a total of approximately $90^{{}^{\circ}}$ beam scanning is available) in the xz plane, also written as the $\varphi =0^{{}^{\circ}}$ plane of reference. To emphasize the efficacy of the coated metasurfaces in enabling the decoupling of the arrays at various beam scan angles, we demonstrate the active VSWR plots and isolation parameter plots for the uncloaked and cloaked Array I in Figure 17 and Figure 18 at scan angles $20^{{}^{\circ}}$ and $30^{{}^{\circ}}$, respectively.

As mentioned above, when Array I is excited, the mutual coupling coefficients (isolation parameters) are observed at ports 2, 4, 6, and 8 and are denoted as $S_{2}$, $S_{4}$, $S_{6}$, and $S_{8}$. Meanwhile, the active VSWRs are plotted at ports 1, 3, 5, and 7 (denoted as VSWR 1, VSWR 3, VSWR 5, and VSWR 7). It is clear from the following figures that, for the uncloaked coupled Array I (see Figure 17a,c and Figure 18a,c), at each of the scan angles, the VSWR plots show degradation in the matching characteristics, and the active isolation coefficients depict high values of the coupling levels, at the resonance frequency of 4.9 GHz. Again, this is a clear indication of the tremendous mutual coupling present between the two uncloaked arrays. From the active VSWR plots and the isolation plots for the cloaked Array I (see Figure 17b,d and Figure 18b,d), at each of the scan angles, it is revealed that the array shows good matching properties and a considerable reduction in mutual coupling at the desired resonance frequency.

**Figure 19:**
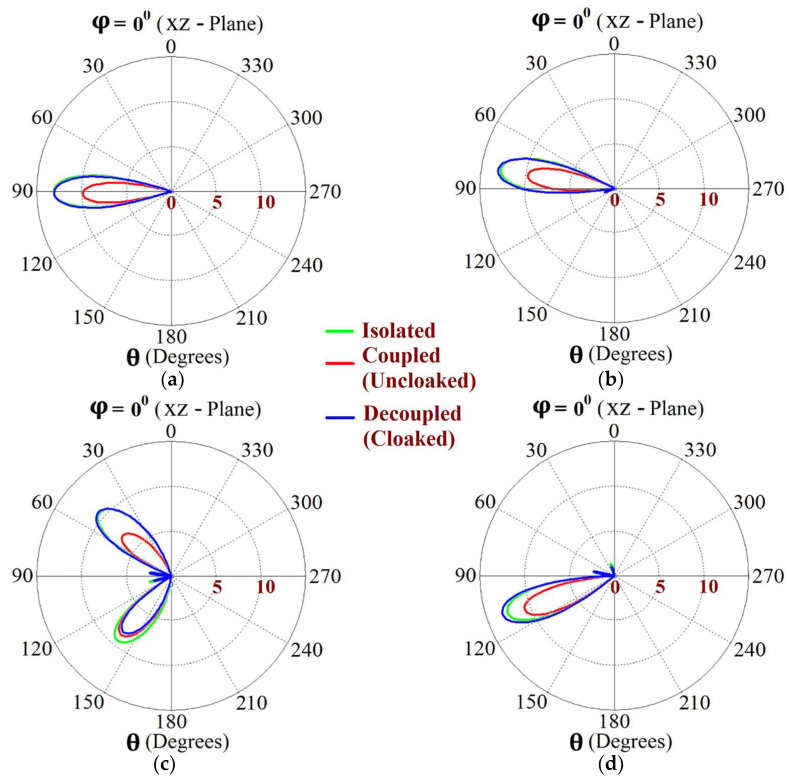
Realized gain plots for Array I ($f_{1}=4.9$ GHz) showing beam scanning at scan angles (**a**) $\theta_{s}=0^{{}^{\circ}}$, (**b**) $\theta_{s}={-10}^{{}^{\circ}}$, (**c**) $\theta_{s}={-45}^{{}^{\circ}}$, and (**d**) $\theta_{s}=20^{{}^{\circ}}$.

**Figure 20:**
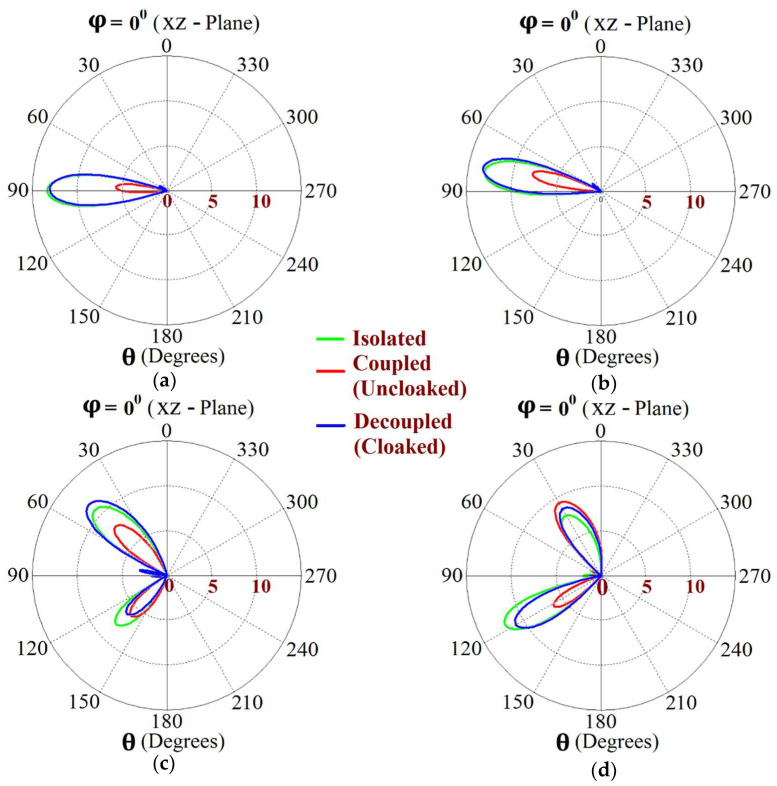
Realized gain plots for Array II ($f_{2}=5.2$ GHz) showing beam scanning at angles (**a**) $\theta_{s}=0^{{}^{\circ}}$, (**b**) $\theta_{s}={-10}^{{}^{\circ}}$, (**c**) $\theta_{s}={-45}^{{}^{\circ}}$, and (**d**) $\theta_{s}=30^{{}^{\circ}}$.

This indicates that the coated metasurface cloaks effectively decouple the two arrays. We further present the polar plots for various beam scanning angles for both Array I and Array II in Figure 19 and Figure 20. It is apparent from the polar plots for both arrays that in the uncloaked (coupled) condition (observe the solid red curves depicted in Figure 19 and Figure 20), a reduction in the main lobe gain as well as some distortion in the pattern itself is present. On the other hand, in the cloaked (decoupled) case (solid blue curves), the metasurface cloaks coating the antenna elements of the arrays are shown to faithfully rehabilitate the realized gain patterns at all the illustrated beam angles. We should also note the side lobes that seem to emerge as the beam steers toward its extreme angles (especially at ${-45}^{{}^{\circ}}$ and $45^{{}^{\circ}}$). Although the side lobes are present, they should not notably affect the main lobe radiation, since a considerable magnitude of gain is still concentrated in the main lobe at this particular angle.

## 5. Circular Patch Antennas

Circularly shaped patches are another popular type of microstrip antenna, especially with regard to developing circularly polarized antenna structures. Similar to the cloak designs detailed in Section 2, we now model corresponding coated metasurface cloaks for these circular patches. With this, we emphasize the versatility of our cloak construct in the sense that these metasurfaces can be tailored to different shapes of the patch antennas.

### 5.1. Decoupling and Cloaking of Two Circularly Shaped Patch Antennas

**Figure 21:**
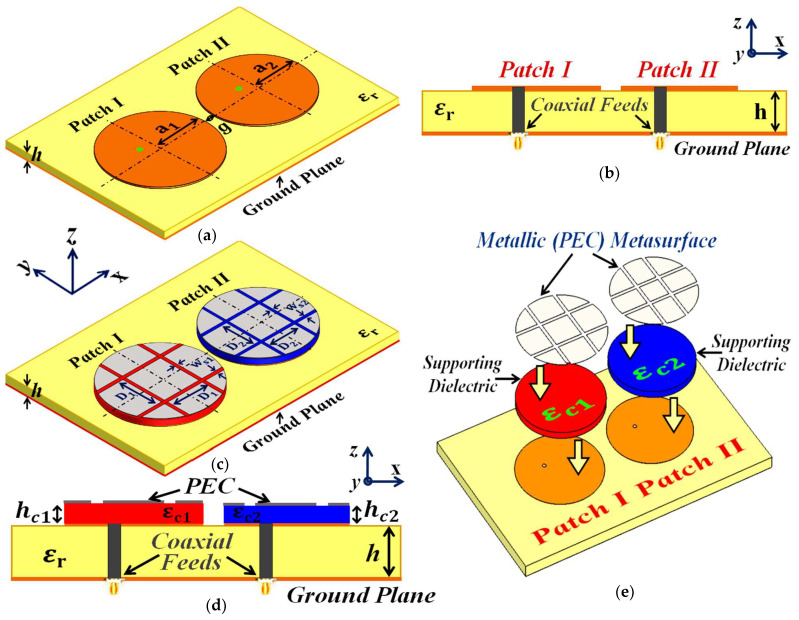
Schematics for (**a**) uncloaked circular Patch I and II, (**b**) cross-sectional side view of the uncloaked coupled patches, (**c**) cloaked Patch I and II, (**d**) side view of the cloaked circular patches, detailing the structural parameters of the coated metasurfaces, and (**e**) unfolded view of the cloak design.

Following the procedure described in Section 2, we consider two coaxially fed circular patch antennas, operating at frequencies $f_{1}=4.5$ GHz and $f_{2}=4.7$ GHz, respectively (refer to Figure 21). The patch antennas are installed on a dielectric substrate with thickness h=1.8 mm and permittivity $\varepsilon_{r}=2.2$. The dimensional values for the uncloaked antennas are as follows (see Figure 21a,b): $a_{1}=11.95$ mm and $a_{2}=11.67$ mm. The antennas are fed diagonally to promote circular polarization in our conceptualized configurations. We adhere to the investigative process mentioned in Section 2 for the rectangular patches, and we record the results related to the three cases: *isolated*, *uncloaked coupled*, and *cloaked decoupled*. These circular patches are located extremely close to each other (g=1 mm $\approx \;0.015\;\lambda_{1}$) and it is obvious that such a tight arrangement leads to a strong mutual coupling effect between the antennas, thereby causing the radiation properties of both the circular patches to deteriorate. Accordingly, to reduce the interference between the patches, we implement the specifically designed metasurface structures (refer to Figure 21c,d) by coating the top surface of each patch antenna with a supporting dielectric material (thickness $h_{c1}=1$ mm and $h_{c2}=1$ mm, and permittivity $\varepsilon_{c1}=24.6$ and $\varepsilon_{c2}=21.2$, for Patch I and Patch II, respectively) and then placing a slotted PEC surface directly onto these dielectrics (slot widths are $w_{s1}=w_{s2}=0.5$ mm and the slot spacing is set as $D_{1}=8.4$ mm and $D_{2}=6.6$ mm). To visualize the stepwise modeling of the coated cloaks, an unfolded view is depicted in Figure 21e. In the previous section, we stated that we have been unable to identify a clear analytical model for the cloak integrated with the patch antenna structure; thus, the optimum values for the design parameters have been determined by conducting an extensive parametric analysis.

The boundary and mesh cell settings that were established in the CST simulation software in order to simulate our designed models for circular patches were as follows. An ‘Open (add space)’ boundary type for all x, y, and z axes and the simulation frequency range of 2 to 7 GHz were selected. The minimum distance of the boundary box from the antenna structure was set at a one-fourth wavelength corresponding to the frequency of 4.5 GHz. The mesh properties were determined by generating the maximum cell using the setting of the ‘cells per wavelength’ value as 36 and 27 for the cases near to the model and far from the model, respectively, and generating the minimum cell by setting the value of the ‘fraction of maximum cell near to the model’ at 21. These settings led to 2,235,156 cells being created by CST for the simulation (all of the aforementioned settings can be tailored to suit specific design necessities).

**Figure 22:**
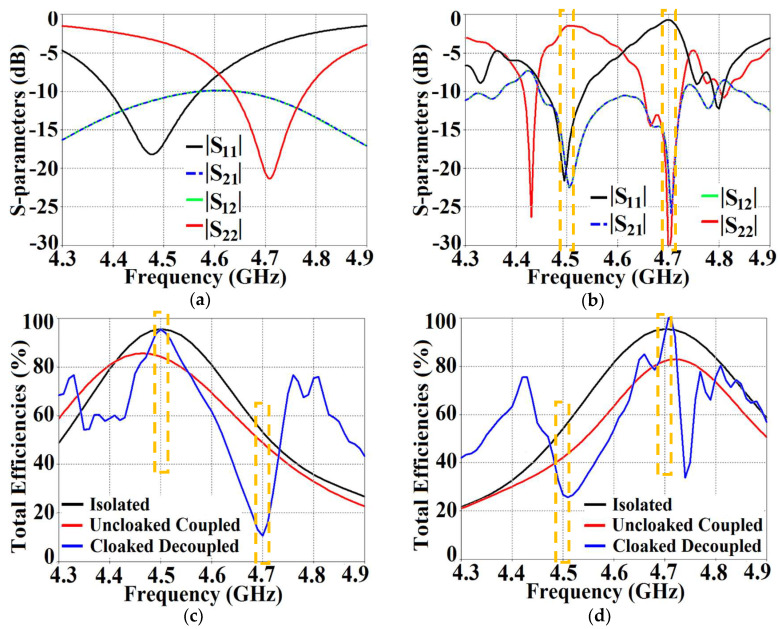
Plots for S-parameters: (**a**) uncloaked coupled and (**b**) cloaked decoupled patch antennas and plots for total efficiencies: (**c**) Patch I is active and (**d**) Patch II is active.

**Figure 23:**
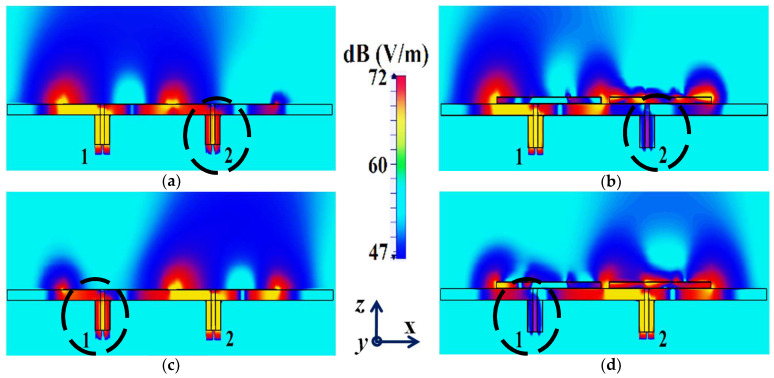
Electric field contours for (**a**) uncloaked coupled and (**b**) cloaked decoupled cases when Patch I is active ($f_{1}=4.5$ GHz), and (**c**) uncloaked coupled and (**d**) cloaked decoupled cases when Patch II is active ($f_{2}=4.7$ GHz).

To establish the decoupling and cloaking effects of the proposed metasurface structures, we present the following simulation results. The S-parameter plots (Figure 22a,b), along with the plots for total efficiencies (Figure 22c,d), clearly show the decoupling action of the coated cloaks, and the E-field contour plots (Figure 23) further corroborate this claim.

It is evident from Figure 22a that the coupling coefficients $\left|S_{12}\right|=\left|S_{21}\right|\approx -10$ dB at $f_{1}$ as well as $f_{2}$, for the uncloaked coupled case, indicating strong mutual coupling between Patch I and II. This mutual coupling magnitude clearly decreases for the cloaked decoupled case (see Figure 22b, wherein a reduction of almost 12 dB and 15 dB in $\left|S_{12}\right|$ and $\left|S_{21}\right|$ is observed at $f_{1}$ and $f_{2}$, respectively). Note that in Figure 22b, $|S_{11}|\approx 0$ dB at frequency $f_{2}$ (indicating that Patch I is decoupled at $f_{2}$), and $\left|S_{22}\right|\approx -1$ dB at frequency $f_{1}$ (indicating that Patch II remains decoupled at $f_{1}$). Moreover, in Figure 22c,d, we also display the total efficiencies of each circular patch antenna in the isolated, uncloaked, and cloaked scenarios. Due to the high levels of mutual coupling between the uncloaked patches, the total efficiency drops by approximately 12% and 15% for Patch I and II, at their resonance frequencies $f_{1}=4.5$ GHz and $f_{2}=4.7$ GHz, respectively (examine the solid red curves in Figure 22c,d). However, the total efficiencies of each of the patches are seen to be recovered for the cloaked case (solid blue curves in Figure 22c,d). Comparing the blue and black curves, we notice that these restored efficiencies are equivalent to the efficiency of the antennas in their isolated condition. Subsequently, we claim that the total efficiency of a cloaked patch antenna remains unchanged at its own resonance frequency but reduces significantly at the neighboring antenna’s operating frequency. In other words, the patterned metasurface does not cause alterations/deterioration in the radiation aspects of the circular patch antenna on which it is coated; instead, its effect is evident at the frequency of the other patch in its vicinity. Additionally, in Figure 23, we present an expanded view of the E-field snapshots for the cloaked and uncloaked circular patches arranged in tight spacing.

**Figure 24:**
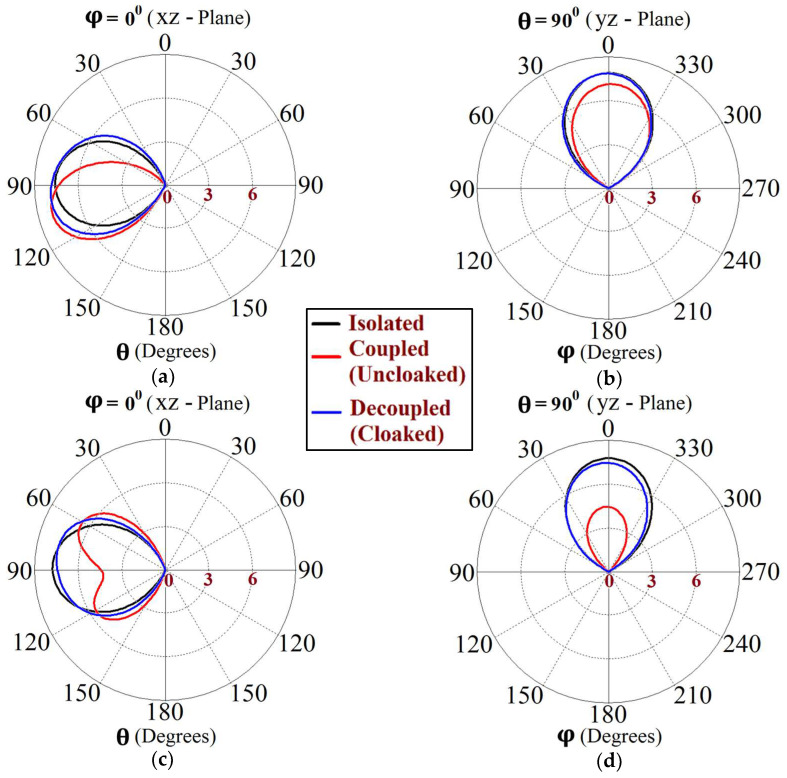
Realized gain patterns at (**a**)$\;\varphi =0^{{}^{\circ}}$, (**b**) $\theta =90^{{}^{\circ}}$ for Patch I (at $f_{1}=4.5$ GHz), and at (**c**)$\;\varphi =0^{{}^{\circ}}$, (**d**) $\theta =90^{{}^{\circ}}$ for Patch II (at $f_{2}=4.7$ GHz).

The E-field contours for Patch I ($f_{1}=4.5$ GHz) in the uncloaked coupled and cloaked decoupled conditions are shown in Figure 23a,b, respectively (here, Patch I is active, whereas Patch II is inactive). Similarly, Figure 23c,d corresponds to the uncloaked coupled and cloaked decoupled cases, respectively, when Patch II is excited and Patch I is passive. Consider Figure 23a; the presence of mutual coupling is obvious due to the power coupling seen from the input port of Patch I (port 1) to the neighboring port 2 (indicated by a high concentration of fields, shown by the red color). On the other hand, when the coated metasurface cloaks are employed (cloaked case, see Figure 23b), they greatly diminish the power coupling from port I to the input port of Patch II (port 2), thus highlighting the decoupling behavior of the coated cloak structure (notice the absence of the concentrated fields at this port). Analogous observations and deductions can be made from the corresponding E-field distributions at $f_{2}=4.7$ GHz, when Patch II is active (refer to Figure 23c,d). To emphasize the cloaking functionality of the metasurfaces in the far-field, polar plots for the realized gain patterns of each patch antenna are presented in Figure 24. The main lobe gain of both the patches in its isolated scenario is around 7.5 dBi. In both the reference planes, for the uncloaked coupled case, a considerable distortion as well as reduction in gain is apparent for Patch I and Patch II (observe the solid red curves in Figure 24). Nevertheless, it is obvious that the specifically tailored metasurfaces faithfully reinstate the gain patterns for both the circular patches (refer to the cloaked decoupled case shown by the solid blue curves in Figure 24), at both the planes of reference.

**Figure 25:**
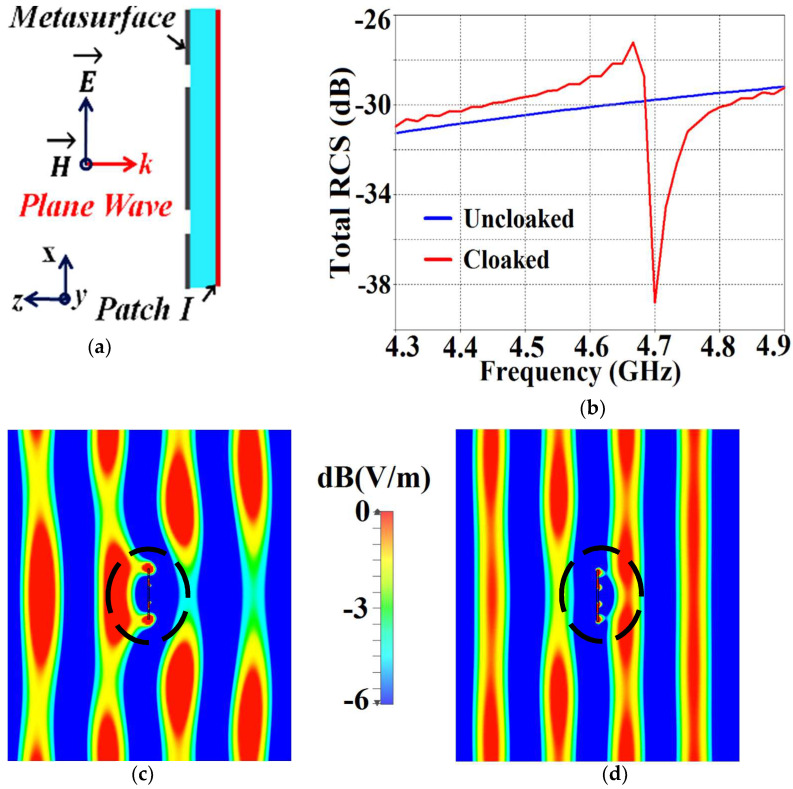
(**a**) Cross-sectional side view, (**b**) total RCS plot for cloaked Patch I, and E-field distributions for cloaked Patch I at (**c**) $f_{1}=4.5$ GHz, (**d**) $f_{2}=4.7$ GHz in presence of a normally incident TM polarized plane wave.

Finally, we demonstrate the scattering cancellation action of our proposed cloaks in Figure 25, wherein cloaked Patch I is subjected to a TM polarized plane wave incident normally to the antenna surface (cross-sectional side view of the design is depicted in Figure 25a). The total RCS plot in Figure 25b shows a considerable reduction in the scattering magnitude (≈8.5 dB decrease is noted for cloaked Patch I when compared to its uncloaked case) at $f_{2}=4.7$ GHz, indicating that the scattering from Patch I is minimized at 4.7 GHz, thus rendering it ‘electromagnetically invisible’ at this frequency. Next, consider the E-field plots depicted in Figure 25c,d. We observe substantial scattering around the edges of Patch I at its resonance frequency, i.e., at $f_{1}=4.5$ GHz (denoted by the concentration of the red-colored region around the patch edges in Figure 25c), whereas, at its cloaking frequency ($f_{2}=4.7$ GHz, which is the resonance frequency of Patch II), the metasurfaces considerably minimize the scattering of the electromagnetic waves around Patch I (absence of red-colored regions around the patch edges, evident from Figure 25d). Similar arguments can be made for cloaked Patch II at its resonance frequency, $f_{2}=4.7$ GHz, and cloaking frequency, $f_{1}=4.5$ GHz (results are not shown for conciseness). As such, we have successfully demonstrated the decoupling and cloaking of our proposed cloaks for the two circular antennas in close proximity.

### 5.2. Cloaking of the Interleaved Circular Patch Antenna Arrays

**Figure 26:**
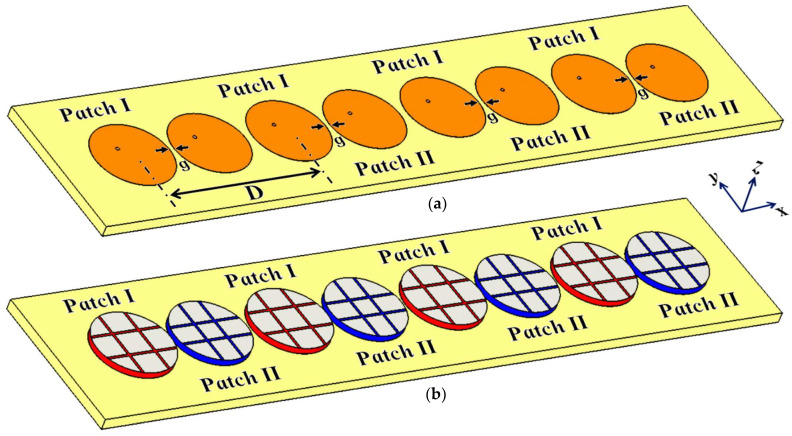
Schematic configurations of (**a**) uncloaked and (**b**) cloaked interleaved circular patch antenna arrays.

The proposed planar metasurface cloak structure is further extended and implemented in a one-dimensional interleaved array of circular microstrip patch antennas. In our conceptualized array designs, four elements each for Patch I and Patch II are considered and these eight patch elements are situated side by side, horizontally, along the x-axis. The schematics for the uncloaked and cloaked interleaved arrays are represented in Figure 26. Array I includes all Patch I antenna elements, and they are spatially arranged at a distance of D=51.2 mm, whereas the elements of Array II comprise the Patch II antennas, with the elements being placed directly beside the antenna elements of Array I at the distance of g. The extremely close proximity of all these patches leads to very strong interference, thus destroying the matching as well as radiation aspects of both the arrays.

For the simulations of the interleaved circular arrays, the mesh properties are set by generating maximum cell using a ‘cells per wavelength’ value of 36 and 24 for the cases near to the model and far from the model, respectively, whereas the minimum cell is generated by setting the value of the ‘fraction of maximum cell near to the model’ as 24. The total number of cells created by the CST software using these settings is 8,583,798.

**Figure 27:**
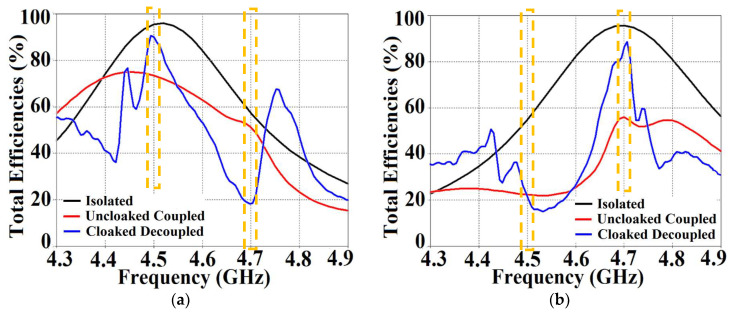
Plots for total efficiencies: (**a**) Array I ($f_{1}=4.5$ GHz) active and (**b**) Array II ($f_{2}=4.7$ GHz) active.

**Figure 28:**
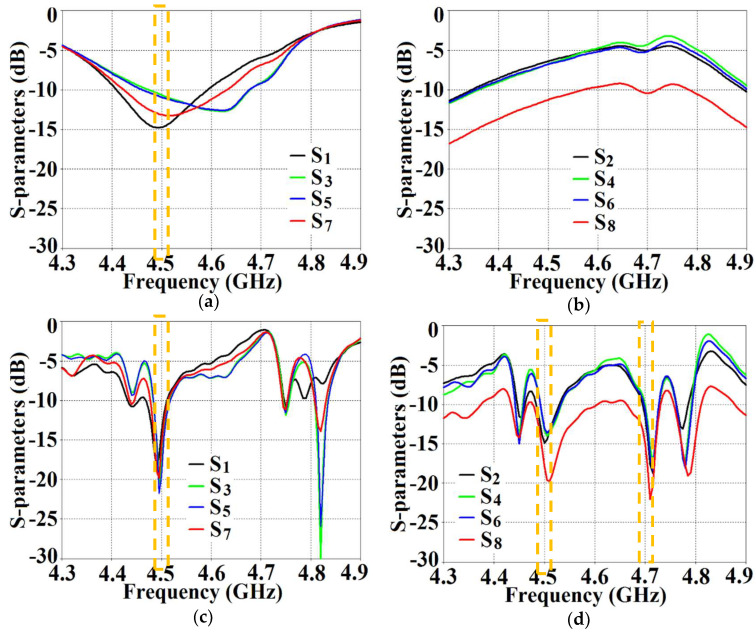
(**a**) Active reflection coefficients, (**b**) active coupling coefficients for uncloaked (coupled) Array I, (**c**) active reflection coefficients, and (**d**) active coupling coefficients for cloaked (decoupled) Array I (resonance frequency–$f_{1}=4.5$ GHz).

**Figure 29:**
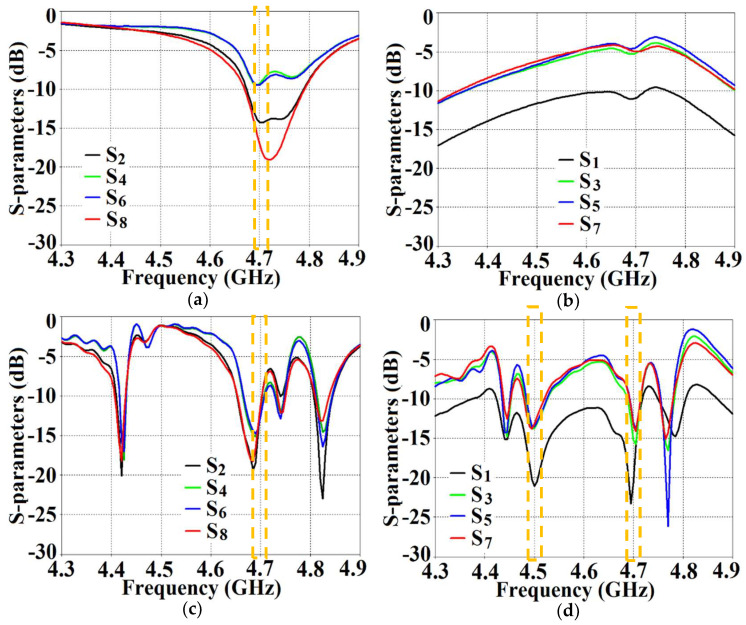
(**a**) Active reflection coefficients, (**b**) active coupling coefficients for uncloaked (coupled) Array II, (**c**) active reflection coefficients, and (**d**) active coupling coefficients for cloaked (decoupled) Array II (resonance frequency–$f_{2}=4.7$ GHz).

From Figure 27, it is obvious that the total efficiency decreases considerably for the uncloaked cases (a reduction of approximately 27% and 42% for Array I and II, respectively, is observed through the red curves in Figure 27). For the cloaked cases, however, the total efficiencies are vastly improved, almost emulating the isolated array scenario (compare the blue curves with the black curves in Figure 27). In an attempt to demonstrate that the arrays are effectively decoupled from each other, we depict the isolation parameters along with the reflection coefficients for the uncloaked arrays and compare them with their cloaked counterparts (Figure 28 and Figure 29). Let us consider Figure 28; the active isolation and reflection coefficients for the uncloaked and cloaked Array I are showcased (the resonance frequency of this array is $f_{1}=4.5$ GHz). When we intend for Array I to be active, ports 1, 3, 5, and 7 are excited. This implies that when Array I is activated, the active reflection coefficients are obtained at ports 1, 3, 5, and 7 (denoted as $S_{1}$, $S_{3}$, $S_{5}$, and $S_{7}$), whereas the active coupling coefficients are abstracted at ports 2, 4, 6, and 8 (denoted as $S_{2}$, $S_{4}$, $S_{6}$, and $S_{8}$). It is evident from Figure 28a that for uncloaked Array I, the matching characteristics are degraded at its own designated operating frequency, $f_{1}=4.5$ GHz. The primary cause of this discrepancy is attributed to the very high levels of mutual coupling arising due to the close placement of the neighboring array elements.

**Figure 30:**
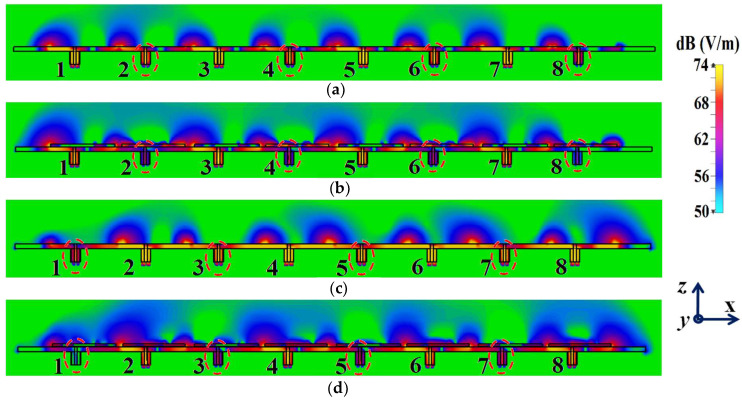
E-field contours: (**a**) uncloaked and (**b**) cloaked patch antenna arrays when Array I is active and (**c**) uncloaked and (**d**) cloaked patch antenna arrays when Array II is active.

The isolation parameters (coupling coefficients) in Figure 28b demonstrate the high mutual coupling magnitudes for the uncloaked array at the frequencies of interest. An interesting behavior that can be noted is that Array I, in the uncloaked form, becomes an unmatched poor radiator at its own resonance frequency due to the power coupling occurring at the neighboring array ports. On the other hand, when the array elements are coated with our proposed metasurface cloaks, Array I is seen to be well matched at 4.5 GHz, as shown in Figure 28c. In addition to this, we observe that Array I is unmatched at the neighboring array’s operating frequency, i.e., at $f_{2}=4.7$ GHz. This is credited to the fact that the coated metasurfaces greatly reduce the mutual coupling levels (see Figure 28d) at each resonance frequency, essentially decoupling the neighboring array elements. Similar arguments and observations can be made through analogy when Array II is active. For this case, ports 2, 4, 6, and 8 are excited, which means that the active reflection coefficients are plotted at these ports (denoted as $S_{2}$, $S_{4}$, $S_{6}$, and $S_{8}$) and the isolation coefficients (denoted as $S_{1}$, $S_{3}$, $S_{5}$, and $S_{7}$) are plotted at ports 1, 3, 5, and 7. The corresponding plots are given in Figure 29. Moreover, we exhibit the E-field contours in Figure 30 (Array I is active and Array II is kept passive, i.e., ports 1, 3, 5, and 7 are excited, in Figure 30a,b; similarly, Array II is active and Array I is passive, i.e., ports 2, 4, 6, and 8 are excited, in Figure 30c,d). For the uncloaked coupled case, interference caused by the coupling between the antenna elements deteriorates the radiation attributes of each array.

When coating the specific metasurfaces onto each corresponding circular patch element (cloaked decoupled scenario), the mutual coupling effects are substantially decreased in the near-field and the restoration of the radiation patterns is noticed in the far-field, thereby greatly improving the overall radiation characteristics of each array.

### 5.3. Beam Scanning

In the above sub-section, we have validated that the engineered metasurfaces enhance the near-field and far-field radiation properties of each patch array in the interleaved system. In brief, the elements of one array are forced to become electromagnetically invisible to and, in turn, decoupled from the elements of the adjoining array. Along with this, the coated metasurfaces facilitate efficient beam scanning capabilities for both the circular patch antenna arrays.

**Figure 31:**
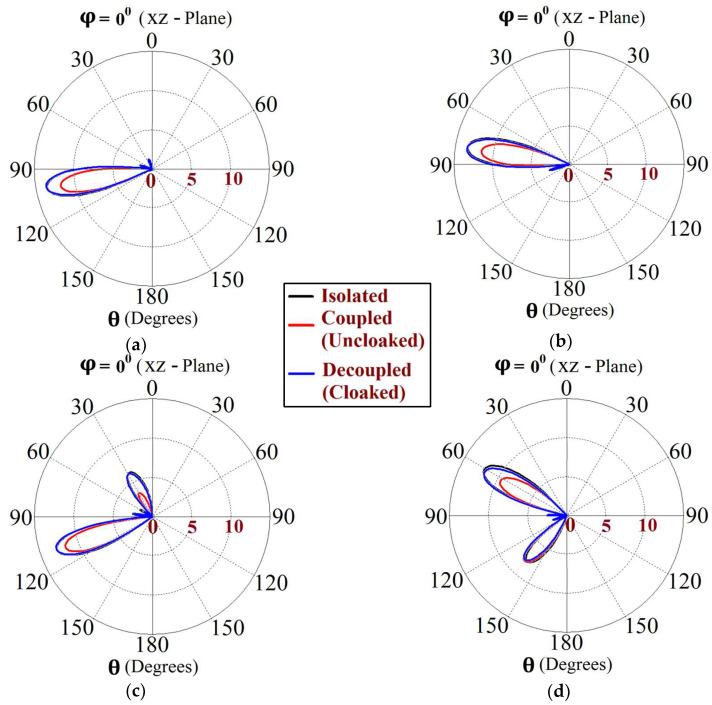
Realized gain polar plots for Array I ($f_{1}=4.5$ GHz) at scan angles (**a**) $\theta_{s}=10^{{}^{\circ}}$, (**b**) $\theta_{s}={-10}^{{}^{\circ}}$, (**c**) $\theta_{s}=20^{{}^{\circ}}$, and (**d**) $\theta_{s}={-30}^{{}^{\circ}}$.

**Figure 32:**
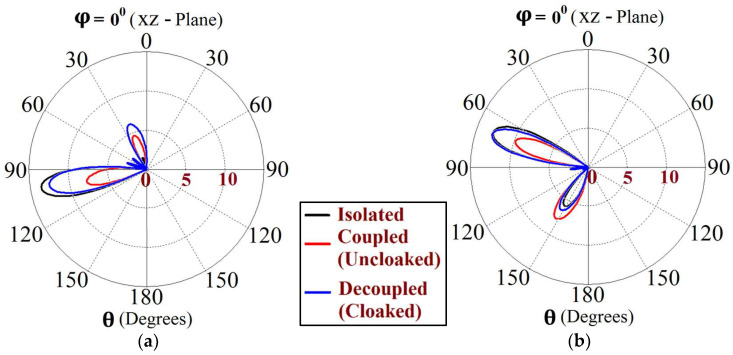
Realized gain polar plots for Array II ($f_{2}=4.7$ GHz) at scan angles (**a**) $\theta_{s}=10^{{}^{\circ}}$ and (**b**) $\theta_{s}={-20}^{{}^{\circ}}$.

Utilizing the commonly known formula for phase shift calculation, we established the range of beam scan angles for our array system. As such, cloaked Array I faithfully scans the angles from $\theta_{s}=-30^{{}^{\circ}}$ to $\theta_{s}=30^{{}^{\circ}}$ and cloaked Array II shows effective beam scanning from the angles $\theta_{s}=-25^{{}^{\circ}}$ to $\theta_{s}=25^{{}^{\circ}}$ in the xz plane. The realized gain polar plots at different scan angles for circular patch Array I and Array II are depicted in Figure 31 and Figure 32, respectively.

The polar plots for both arrays clearly indicate that the cloaks coating the antenna elements successfully restore the realized gain patterns at all the illustrated scan angles. The presence of grating lobes is observed at some of the scan angles; even so, the main lobe gain is significantly high as compared to these side lobes, at each of these angles.

Finally, we present a comparison between our proposed planar coated metasurface construct and other cloaking approaches, as well as a few alternative decoupling approaches, for two antennas operating in distinct frequency bands. The results are highlighted in Table 1.

Please note that the values in the ‘Coupling Reduction’ and ‘Main Lobe Gain Enhancement’ columns are given for the decoupled cases, when compared to their coupled counterparts. Almost all of the referred works achieved considerable amounts of isolation enhancement (decoupling) between the antenna elements, especially [25,27,40,41]. Moreover, some of these works targeted antenna array applications and showed the good suppression of cross-band scattering and coupling for the intended frequency range. Nevertheless, there are multiple aspects that set our proposed cloaking mechanism apart from the reported decoupling methods. Firstly, our cloaks possess a very simple planar design and are coated directly onto each antenna surface. In addition to achieving excellent decoupling and gain enhancements for the rectangular and circular patch antennas at each intended frequency, our cloaks are capable of suppressing scattering from the interleaved arrays as well (at predetermined frequencies). Another clear distinction point is the antenna element separation. In our methodology, the antennas are placed very close to each other (sub-wavelength separation), confirming that the mutual coupling levels in such a scenario are bound to be extremely high. Nevertheless, our designed cloaks substantially reduce the interference effects in the near-field as well as the far-field (not only for two elements but for the array configurations as well). However, our structures have a very narrow band response and it is our intention to improve the impedance bandwidth of our cloaks by possibly devising a wideband cloaking system in the near future.

## 6. Conclusions and Future Directions

We have proposed a planar metasurface design for the cloaking and decoupling of rectangular and circular patch antennas, along with their interleaved phased arrays, as an extension. The numerous simulation results presented in the paper serve to validate our claim that these specifically designed metasurfaces mitigate the mutual coupling effect when two patches are placed very close to each other. The metasurface cloaks not only decouple the antennas in the near-field but also favorably restore their radiation characteristics in the far-field, such that it seems that each antenna is radiating independently. In other words, the cloaks facilitate unhindered antenna radiation so as to emulate its radiation performance in an isolated environment (absence of other antennas/radiators). The crucial aspect of any cloaking structure is that it should not only improve the near-field characteristics (results depicted through the S-parameter plots, total efficiencies, and near-field E-field distribution plots), but also reinstate the radiation properties of the antenna in the far-field (this is demonstrated through the various polar plots). Along with this, we have also showcased the total radar cross-section (RCS) plots and highlighted the scattering cancellation capability of the cloaks (using E-field plots) to accentuate the cloaking functionality of the metasurface. In each of the patch antenna configurations described in the manuscript, the concept of decoupling and cloaking was further extended to interleaved one dimensional antenna array systems, where we have demonstrated the decoupling of phased antenna arrays for a wide range of beam scanning angles. Subsequently, we have presented printed antenna systems that can seamlessly accommodate two separate arrays (different frequencies) with virtually no increase in the spatial or dimensional area, essentially promoting frequency diversity, which we believe will be capable of handling ever-increasing network capacity requirements and also possibly lower the operating costs. The extensibility of our coated cloaks, demonstrated by their ability to cloak both rectangular and circular patches (albeit using distinct dimensional parameters for each corresponding cloak), leads us to believe that this design could be modified to accomplish the cloaking of other printed antenna configurations as well. Owing to the simplicity of the structure, the fabrication of the cloak is quite feasible. It is based on the fact that microstrip antennas are one of the most popular types of printed antenna, and our designed systems can easily find application in today’s world of wireless communication. For instance, satellite communication primarily requires circularly polarized radiation patterns, which can be realized using either square (rectangular) or circular patch microstrip antennas. Applications could also be found in the fields of radio frequency identification (RFID), mobile communication (especially WiMax-worldwide interoperability for microwave access-based communication equipment), and even healthcare. Although our present work focuses on the 5G applications of the designed antennas in the ‘C-band’, our investigations have revealed that the proposed cloak designs can very well be configured to any desired frequency range (even beyond the C-band spectrum) and thus can potentially lead to possible applications on the 3G and 4G/LTE platforms as well.

As an immediate insight into our future work, we are working on obtaining the necessary equipment to support our claims with experimental verifications through fabricated prototypes. We also plan to investigate alternative arrangements of the interleaved array system of the rectangular and circular patches to obtain a much wider range of beam scan angles. To improve the bandwidth performance of our cloaking structures, we are looking into the possibility of utilizing stacked multi-layered (at least two layers) planar metasurfaces (inspired by the wideband cloaking method described in [32]). We are also considering the prospect of exploiting the reconfigurable metasurfaces concept, to potentially develop an ‘intelligent’ antenna system.

## Figures and Tables

**Table 1:** Comparison of our proposed method with other decoupling approaches.

Ref.	Decoupling Approach	Frequency Bands (GHz)	Array Application	Antenna Element Separation (mm)	Reflection Coefficient (dB)	Coupling Reduction (dB)	Main Lobe Gain Enhancement (dB)
[22]	Cloaking (Circular Cloaks)	LF ^1^: 3.07 HF ^2^: 3.33	Not addressed	10	LF ^1^: −12 (C ^3^) −17 (Dc ^4^) HF ^2^: −11.5 (C ^3^) −16 (Dc ^4^)	Not addressed	LF ^1^: ~2 HF ^2^: ~1.5 (Gain pattern restored)
[25]	Cloaking (Elliptical Cloaks)	LF ^1^: 3 HF ^2^: 3.33	Not addressed	16	LF ^1^: −19 (C ^3^) −20 (Dc ^4^) HF ^2^: −12 (C ^3^) −21 (Dc ^4^)	LF ^1^: ~25.5 HF ^2^: ~29.5	LF ^1^: ~1 HF ^2^: ~1 (Gain pattern restored)
[26]	Cloaking (Circular Cloaks)	LF ^1^: 0.92 HF ^2^: 3.2	Not addressed	36	At LF ^1^: −14 At HF ^2^: −20	Not addressed	At HF ^2^: ~5 (Gain pattern restored)
[27]	Cloaking (Elliptical Cloaks)	LF ^1^: 4.5 HF ^2^: 5.5	Possible but not addressed	3	LF ^1^: −18 (C ^3^) −30 (Dc ^4^) HF ^2^: −12 (C ^3^) −30 (Dc ^4^)	LF ^1^: ~20 HF ^2^: ~20	LF ^1^: ~1.32 HF ^2^: ~3 (Gain pattern restored)
[29]	Cloaking (Circular Cloaks)	LF ^1^: 0.79–0.86 HF ^2^: 1.9–2.2	Not addressed	~36.3	LF ^1^: −25 (C ^3^) −18 (Dc ^4^) HF ^2^: −8 (C ^3^) −17.5 (Dc ^4^)	At HF ^2^: ~10	At HF ^2^: ~2–3 (Gain pattern restored)
[30]	Cloaking (Circular Cloaks)	LF ^1^: 0.69–0.96 HF ^2^: 1.71–2.71	LF ^1^ antenna placed over 2 × 3 HF ^2^ array	~20	Not addressed	Not addressed	For HF ^2^ array: ~4
[37]	C-PDDN ^5^	LF ^1^: 2.3–2.4 HF ^2^: 2.4–2.48	Possible but not addressed	96.5	LF ^1^: −20 (C ^3^) −40 (Dc ^4^) HF ^2^: −15 (C ^3^) −20 (Dc ^4^)	LF ^1^: ~21 HF ^2^: ~21	Not addressed
[38]	FSS ^6^ Radiator	LF ^1^: 1.8–2.7 HF ^2^: 3.3–3.8	LF ^1^ antenna placed over 2 × 2 HF ^2^ array	~25	At LF ^1^: −22.5 (Dc ^4^) For HF ^2^ array: −27.5 (Dc ^4^)	At LF ^1^: ~25 At HF ^2^: ~21	Gain enhancement not addressed. Peak gain: At LF ^1^: ~7 For HF ^2^ array: ~9
[40]	Slot Loading	LF ^1^: 0.69–0.96 HF ^2^: 1.7–2.4	LF ^1^ antenna placed over 2 × 2 HF ^2^ array	~33	At LF ^1^: −25 (Dc ^4^) For HF ^2^ array: −30 (Dc ^4^)	At LF ^1^: ~30 At HF ^2^: ~30	Gain enhancement not addressed. Peak gain: At LF ^1^: ~8 For HF ^2^ array: ~12
[41]	2.5 D Cloak Loading	LF ^1^: 1.64–2.56 HF ^2^: 4.4–5.0	LF ^1^ antenna placed over HF ^2^ array	40	At LF ^1^: −25 (Dc ^4^) For HF ^2^ array: −20 (Dc ^4^)	At LF ^1^: ~25 At HF ^2^: ~25	Peak gain: At LF ^1^: ~7 At HF ^2^: ~7.2
PW ^7^	Cloaking (Planar Cloaks)	LF ^1^: 4.9 HF ^2^: 5.2	Interleaved array (side-by-side antenna placement)	2	LF ^1^ array:>−10 (C ^3^) ~−15 (Dc ^4^) HF ^2^ array:>−10 (C ^3^) ~−12 (Dc ^4^)	At LF ^1^ array:~12.5 At HF ^2^ array:~12.5	At LF ^1^ array: ~3.2 At HF ^2^ array: ~8.3 (gain pattern for each array is restored)
PW ^7^	Cloaking (Planar Cloaks)	LF ^1^: 4.5 HF ^2^: 4.7	Interleaved array (side-by-side antenna placement)	1.5	LF ^1^ array:−12 (C ^3^) −20 (Dc ^4^) HF ^2^ array:−11 (C ^3^) −18 (Dc ^4^)	At LF ^1^ array: ~8 At HF ^2^ array: ~12	At LF ^1^ array: ~3.7 At HF ^2^ array: ~6.8 (gain pattern for each array is restored)

^1^ Lower frequency, ^2^ higher frequency, ^3^ coupled, ^4^ decoupled, ^5^ cascaded power dividing decoupling network, ^6^ frequency-selective surface, ^7^ proposed work.

## Data Availability

Data are contained within the article.
